# The genome of *Diuraphis noxia*, a global aphid pest of small grains

**DOI:** 10.1186/s12864-015-1525-1

**Published:** 2015-06-05

**Authors:** Scott J Nicholson, Michael L Nickerson, Michael Dean, Yan Song, Peter R Hoyt, Hwanseok Rhee, Changhoon Kim, Gary J Puterka

**Affiliations:** USDA Agricultural Research Service, Stillwater, OK 74075 USA; National Institutes of Health, National Cancer Institute, Bethesda, MD 20892 USA; Department of Molecular Biology and Biochemistry, Oklahoma State University, Stillwater, OK 74078 USA; Axeq Technologies, Rockville, MD 20850 USA

**Keywords:** *Diuraphis noxia*, Russian wheat aphid, Plant-insect interactions, Phytotoxic, Aphid, Genome

## Abstract

**Background:**

The Russian wheat aphid, *Diuraphis noxia* Kurdjumov, is one of the most important pests of small grains throughout the temperate regions of the world. This phytotoxic aphid causes severe systemic damage symptoms in wheat, barley, and other small grains as a direct result of the salivary proteins it injects into the plant while feeding.

**Results:**

We sequenced and *de novo* assembled the genome of *D. noxia* Biotype 2, the strain most virulent to resistance genes in wheat. The assembled genomic scaffolds span 393 MB, equivalent to 93% of its 421 MB genome, and contains 19,097 genes. *D. noxia* has the most AT-rich insect genome sequenced to date (70.9%), with a bimodal CpG(_O/E_) distribution and a complete set of methylation related genes. The *D. noxia* genome displays a widespread, extensive reduction in the number of genes per ortholog group, including defensive, detoxification, chemosensory, and sugar transporter groups in comparison to the *Acyrthosiphon pisum* genome, including a 65% reduction in chemoreceptor genes. Thirty of 34 known *D. noxia* salivary genes were found in this assembly. These genes exhibited less homology with those salivary genes commonly expressed in insect saliva, such as glucose dehydrogenase and trehalase, yet greater conservation among genes that are expressed in *D. noxia* saliva but not detected in the saliva of other insects. Genes involved in insecticide activity and endosymbiont-derived genes were also found, as well as genes involved in virus transmission, although *D. noxia* is not a viral vector.

**Conclusions:**

This genome is the second sequenced aphid genome, and the first of a phytotoxic insect. *D. noxia*’s reduced gene content of may reflect the influence of phytotoxic feeding in shaping the *D. noxia* genome, and in turn in broadening its host range. The presence of methylation-related genes, including cytosine methylation, is consistent with other parthenogenetic and polyphenic insects. The *D. noxia* genome will provide an important contrast to the *A. pisum* genome and advance functional and comparative genomics of insects and other organisms.

**Electronic supplementary material:**

The online version of this article (doi:10.1186/s12864-015-1525-1) contains supplementary material, which is available to authorized users.

## Background

Aphids rapidly radiated as parasites of flowering plants following the spread and diversification of angiosperms 80 to 150 million years ago [1,2]. From that point forward, aphids developed host-specific relationships through use of specialized piercing-sucking mouth parts that penetrate plant tissues to feed upon phloem sap. Key to this feeding process is the injection of saliva which modulates plant defenses [3,4]. More than 5,000 aphid species exist, and over 100 species are economically important crop pests [5]. The Russian wheat aphid, *Diuraphis noxia* Kurdjumov, gained recognition as a global pest of wheat when it rapidly expanded its range from Central Asia and Europe [6] to most of the wheat producing continents over a 15 year period beginning in the early 1970s [7,8]. Losses in wheat exceeded $986 million over the first 10 years after this aphid invaded the United States in 1986 [9].

The genome of the pea aphid, *Acyrthosiphon pisum*, is currently the sole genomic model available for study of aphid biology, genetics, and aphid-plant interactions [10]. *A. pisum* and *D. noxia* share many biological traits common to the family Aphididae. However, a phylogenetic analysis of *Buchnera aphidicola* sequences from a large sample of aphid species indicated that *D. noxia* diverged early in the evolution of the tribe Macrosiphini in the subfamily Aphidinae [11], which includes *A. pisum*, to develop unique host preferences and feeding relationships. The majority of aphids, including *A. pisum*, cause minor damage to their host plants by imposing a metabolic burden through constant removal of phloem sap [3,4,12,13]. In contrast, *D. noxia* represents an economically important group of aphids whose saliva induces rapid, direct, and systemic phytotoxic effects in the host plant, including chlorosis, loss of turgor, abnormal leaf growth, and necrosis [3,14]. *A. pisum* is a well known vector of plant viruses [15] and expanded its host range in legumes through the development of host races that are specific to a plant species [16,17]. *D. noxia* is not a vector of plant viruses [18], and feeds upon over 140 species in 40 genera of graminaceous plants including wheat and barley [19]. This species demonstrates the ability to develop virulent strains, termed biotypes, in response to single gene-based resistance in wheat [20-22] which follows a virulence gene-resistance gene model often associated with plant-parasite relationships [23-25]. Currently, no additional *D. noxia-*resistant wheat cultivars have been released since 2003, when *D. noxia* Biotype 2 overcame *Dn*4 gene-based resistance in wheat. Although *D. noxia* is generally known to reproduce sexually, Biotype 2 is strictly parthenogenetic and a highly successful isofemale component of the genotypically diverse population in the United States [24].

We present this draft version of the *D. noxia* genome as the first crucial step in the study of phytotoxic aphid-plant interactions and the virulence genes that overcome resistance genes in wheat. The advancement of a phytotoxic aphid model will increase the understanding of how virulence genes and their products neutralize host plant resistance genes and the underlying mechanisms of the different aphid-host interactions. Further, the *D. noxia* genome provides an exceptional contrast to *A. pisum* that will facilitate functional and comparative genomics studies of aphids and advance the science of how insects adapted to perform their specialized roles in the environment.

## Results and discussion

### Genome assembly

Genomic DNA from a parthenogenetic isofemale line of *D. noxia* Biotype 2 was sequenced using an Illumina Hi-Seq 2000 and quality filtered, resulting in 496,145,410 paired end reads (read length 101 bp, fragment length 223 bp), 475,489,616 individual 2.5 kb mated-pair reads and two independent 8 kb mated pair libraries with 369,474,230 individual reads that were used for de-novo assembly by Allpaths-LG (Table 1). Final genome coverage was 104X, and the assembly consisted of 49,379 contigs (>1,000 bp, N_50_ = 12,578 bases) and 5,641 scaffolds (N_50_ = 397,774 bases) (Table 2). The genomic scaffolds covered 393,024,634 bases, including 98,530,005 Ns representing unsequenced gaps. RNAseq analysis (Illumina Hi-Seq 2000) was performed using whole-body RNA extracted from the same colony and de novo assembled (Trinity), resulting in 85,990 assembled contigs (≥200 bp, N_50_ = 2,863 bp) (Table 2). The *D. noxia* genome consists of five holocentric chromosomes totaling 421 MB (1C) [26,27] of which our assembly spans 93% (393 MB) including gaps. The *D. noxia* genome as measured by flow cytometry is 18.6% smaller than the genome of the model aphid *A. pisum* (517 MB).

**Table 1:** **Quality-filtered and Buchnera-filtered sequencing data used to assemble the** ***D. noxia*** **biotype 2 genome**

**Sample Name**	**Number of reads (x10** ^**6**^ **)**	**Read Length (BP)**	**Fragment length (BP)**	**Total coverage (GBP)**
**Paired-End**	496.1	2 x 101	223	50.12
**Mated-Pair 2.5 kb**	475.5	2 x 101	2603	48.05
**RWA MP 8 kb**	369.5	2 x 101	8898	37.33
**RWA RNA-seq**	251.8	2 x 101	172	42.92

Reads were filtered prior to assembly so that for a pair of PE reads, each read should have 90% of bases with base quality better than or equal to Q_20_.

**Table 2:** ***D. noxia*** **De novo genome assembly statistics**

	***D. noxia*** **WGS**	***D. noxia*** **RNA-seq**
**Number of Contigs**	49,379(≥1000 bp)	85,990 (≥200 bp)
**Number of Scaffolds**	5,641	NA
**Total Contig Length**	293,543,926	99,888,423
**Total Scaffold Length**	393,024,634	NA
**Contig N**_**50**_	12,578	2,863
**Scaffold N**_**50**_	397,774	NA
**Largest Contig (bp)**	147,337	32,914
**Largest Scaffold (bp)**	2,142,037	NA
**GC/AT percentage**	29.06% GC/70.94% AT	32.8% GC/67.2% AT
**CEGMA genes (complete/partial)**	86.3%/94.4%	99.6%/99.6%

De novo genome assembly performed by Allpaths-LG, de novo transcriptome assembly performed by Trinity.

The *D. noxia* genome is composed of 29.1% G + C and 70.9% A + T which is the lowest G + C percentage of any currently-assembled insect genome including *A. pisum* (29.6% G + C) [10]. The median G + C composition of all identified *D. noxia* transcripts, discussed below, is 39.3% with a range of 21.4% to 72.0%, compared with medians of 38.8% in *A. pisum* [10] and 38.6% in *Apis mellifera* [28]. The high A + T compositions of *D. noxia* and *A. pisum* contradict the hypothesized positive correlation between insect genome size and A + T content [29].

The rate of single nucleotide polymorphisms within the *D. noxia* assembly was measured at 0.45%, and is most likely attributable to the heterozygous chromosomal state that is perpetuated by the strict parthenogenetic reproduction observed in *D. noxia* Biotype 2 [24]. The experimental population consisted of the offspring of one female aphid, therefore, chromosomal heterozygosity was preserved in this clonal population. *D. noxia*’s SNP rate is similar to that of other insects [30,31], is beneath the ≤1% threshold of typical allelic variance [10], and confirms the existence of chromosomal heterozygosity in Biotype 2, as has been noted in other invasive clonal aphid lineages [32].

The telomeric sequence (TTAGG_N_) common to insects [10,33,34] was not found in *D. noxia,* supporting the findings of Novotna *et al.* [27], who were unable to detect common telomere sequences in this aphid by fluorescence *in-situ* hybridization (FISH) analysis. However, RNAseq read mapping revealed the expression of six telomere-related proteins present in the *D. noxia* genome (Additional file 1: Table S1), suggesting the existence of modified telomeric repeat sequences. The lack of classical telomeric sequences is not surprising as altered telomeric sequences, or the substitution of retrotransposons and satellite repeats, have been reported in several other unrelated insect species [33-36].

The completeness of the *D. noxia* genome was assessed using a hidden Markov model (HMM)-based search (CEGMA) of the genome scaffolds and assembled transcripts to identify individual members of the Conserved Eukaryotic Gene (CEG, n = 248) set, which are expected to be present in all eukaryotes [37]. CEGMA analysis determined that the *D. noxia* genome assembly contains 94.4% of the total CEG set, including 214 complete and 20 partial CEGS, for a total of 234 identified CEGS. CEGMA analysis of the predicted *D. noxia* transcriptome found 247 complete CEGs, or 99.6% of the CEG set (Table 2). The identification of 94% of CEGs strongly supports our estimated genome assembly of 93% with gaps likely due to repetitive regions that are recalcitrant to assembly [31].

### CpG dinucleotides and cytosine methylation

Cytosine methylation is the definitive mark of epigenetic regulation in eukaryotes, but occurs only in the CpG context in insects [38]. While DNA methylation is present in most insects, it is only rarely observed among the holometabolous insect orders Coleoptera and Diptera, and is suspected to be undergoing evolutionary deletion in these orders [39,40]. Among hemipteran insects, *A. pisum* and *Pediculus humanus* each display evidence of cytosine methylation, but *P. humanus* lacks the *de novo* methyltransferase Dnmt3 [38]. Epigenetic mechanisms are responsible for the regulation of polyphenism in insects [41,42] and the existence of these mechanisms is signified by a bimodal distribution of observed/expected CpG ratios (CpG_(O/E)_) [38,42,43]. Bimodally-distributed CpG_(O/E)_ ratios indicate the existence of heavily- and lightly-methylated gene groups, with low and high CpG_(O/E)_ ratios, respectively. Divergence of CpG_(O/E)_ ratios in each gene group is due to depletion of CpG dinucleotides over time by the spontaneous deamination of methylcytosine and resulting conversion to thymidine, a process which occurs in all eukaryotes [42-45].

**Figure 1:**
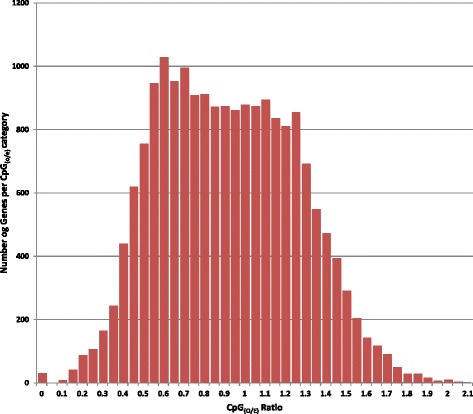
The distribution of observed/expected CpG dinucleotide ratios among predicted *D. Noxia* transcripts. CpG_(O/E)_ distributions of all predicted transcripts were determined according to the equation CpG_(O/E)_ = CpG frequency / [C frequency x G frequency]. The CpG_(O/E)_ distribution of *D. Noxia* is bimodal. Y = number of sequences per category, X = CpG_(O/E)_ ratio category (0.05 per category).

The median CpG composition of *D. noxia* genomic contigs is 2.56% (ranging from 0.0-13.7%) and of predicted transcripts is 2.82% (ranging from 0.0%-19.7%) (Additional file 2: Table S2). Genomic contigs contained 15,827,576 CpG dinucleotides, and predicted transcripts contained 1,588,448 CpG dinucleotides. Analysis of CpG_(O/E)_ ratios revealed a bimodal distribution (kurtosis = −1.54, skewedness = 0.51) with peaks at 0.60 and 1.10 (Figure 1) which is notably similar to those of *A. pisum* [42], *Locusta migratoria* [30], and *Apis mellifera* [43]. In contrast, the unimodal distributions of the holometabolous species *Drosophila melanogaster*, *Nasonia vitripennis, Bombyx mori, Daphnia pulex,* and *Tribolium castaneum* [38,42] indicate the gradual elimination of methylated CpG dinucleotides over time, or the existence of a mechanism which preserves CpG dinucleotides [38]. Peak height comparison reveals that low-CpG_(O/E)_ genes are more abundant than high-CpG_(O/E)_ genes in both *D. noxia* and *A. pisum*, while the opposite is true in all other examined insects, which are obligately holocyclic and are not morphologically polyphenic [38]. The bimodality of CpG_(O/E)_ ratios in *D. noxia* is supported by our finding of a complete DNA methylation gene repertoire, and indicates that DNA methylation is an important regulatory mechanism of gene expression in *D. noxia* [38,42,43].

### Transposable and repetitive elements

Transposable and repetitive elements are a major component of most insect genomes, although the proportion of the genome occupied by these elements varies by species. Transposable and repetitive element expansions lead to increases in genome size, and may be responsible for speciation events among isolated populations [46-48]. Likewise, reductions in genomic repetitive element proportions are observed in small genomes, potentially as a result of reductions of inefficient genomic elements while maintaining a functional gene complement [31,34,49].

Transposable and repetitive elements make up 15.31% of the assembled *D. noxia* genome (Table 3) which is median to the known range for Hemipterans (1% (*P. humanus*) - 38% (*A. pisum*)) [10,34] and of other insect species as well (0.61% to 60%) [30,31]. Analysis of repetitive elements in *D. noxia* determined that most repeats are unclassified repetitive elements or DNA elements (5.02% and 4.17% of the genome respectively) followed by simple repeats (3.89%), SINEs (0.87%), low complexity repeats (0.54%), LINEs and LTR elements (0.79%), and small RNA elements and satellites (0.03%). The nearly 50% reduction in repetitive element percentage in the *D. noxia* assembly is remarkable when compared with *A. pisum* which has an assembled genome only 15.3% larger than *D. noxia*. High repetitive element percentages correlate with increases in genome size, but not with increased gene content [31,49]. Analyses of genome size *versus* directly measured repetitive element content among 12 insect species [10,30,31,34,50-56] suggests an exponential correlation (y = 14.56ln(x) - 60.50, R^2^ = 0.742) (Additional file 3: Figure S1), wherein *D. noxia*’s repetitive element percentage is more consistent with a smaller genome size.

**Table 3:** **Summary of transposable and repetitive elements in the** ***D. noxia*** **genome**

**Element type**		**Number of elements**	**Length occupied**	**Percentage of genome** ^**A**^	**Percentage of genome** ^**B**^
**SINEs**		10,729	2,578,098	0.65	0.87
	ALUs	0	0	0	0
	MIRs	1	58	0	0
**LINEs**		8,415	1,047,278	0.27	0.35
	LINE1	623	33,258	0.01	0.01
	LINE2	2,705	206,618	0.05	0.07
	L3/CR1	695	95,452	0.02	0.03
**LTR elements**		6,338	1,319,571	0.33	0.44
	ERVL	61	3,546	0	0
	ERV_classI	443	24,913	0.01	0.01
	ERV_classII	359	17,479	0	0
**DNA elements**		71,820	12,373,070	3.13	4.17
	hAT-Charlie	3,564	466,416	0.12	0.16
	TcMar-Tigger	139	15,935	0	0
**Unclassified:**		70,950	14,872,045	3.76	5.02
**Total Interspersed Repeats**		**NA**	**32,190,062**	**8.14**	**10.85**
**Small RNA**		256	19,670	0	0.01
**Satellites**		628	48,648	0.01	0.02
**Simple repeats:**		246,285	11,528,041	2.92	3.89
**Low complexity:**		31,355	1,595,105	0.40	0.54
**Total:**			**45,381,526**	**11.47**	**15.31**

^A^Percentage of total genome, including N-containing scaffold gaps, occupied by the indicated transposable and repeat elements. ^B^Percentage of total genome, excluding N-containing scaffold gaps, occupied by the indicated transposable and repeat elements.

### Gene and protein model prediction

Gene and protein models were derived from evidence-based predictions using MAKER software after initially assessing gene predictions from Augustus and MAKER. Augustus predicted 32,440 proteins using Trinity-assembled *D. noxia* transcripts as EST evidence, and 25,003 proteins using *A. pisum* transcripts (NCBI refseq) as EST evidence. MAKER predicted 19,097 genes using *D. noxia* RNAseq data as EST evidence, the NCBI pea aphid protein database as supporting data, and the full RepBase repeat database to identify and mask repetitive elements (Table 4). Gene models predicted by AUGUSTUS were more abundant but significantly shorter than MAKER-predicted models, and in some cases, single genes were classified as multiple genes. We chose the more conservative MAKER-derived gene model set for all subsequent analyses. The total length of the MAKER-predicted transcriptome was 25,135,138 bases, or 5.97% of the genome, within the low end of the range (1.6 -19.4%) for sequenced insect genomes [31,54]. PFAM analysis of the *D. noxia* protein set identified 5,799 proteins harboring 27,262 known PFAM domains (Additional file 4: Table S3). RNAseq mapping to the predicted transcript set revealed that 3,608 genes (18.9%) were not detectably expressed (Additional file 1: Table S1), while a BLASTN comparison (E ≤ 1.0^−15^) of Trinity-assembled transcripts *vs.* MAKER-predicted transcripts determined that 3,313 (17.3%) predicted transcripts were absent from the RNAseq data. The absence of detected transcription of a portion of *D. noxia* genes indicates that a number of genes may be expressed only under certain environmental or nutritional stresses outside the host plant/environmental conditions we used to rear the insects, or that gene expression occurred at low frequencies in specific tissues, and are best addressed specifically through conducting tissue-specific RNAseq experiments.

**Table 4:** **Evidence-based and** ***ab initio*** **gene and protein predictions**

**Gene modeling software**	**Prediction method**	**Transcript/protein predictions**	**Ave./median protein length**	**Ave./median transcript length**	**Longest/shortest transcript**	**Total number of amino acids**	**PFAM motifs**
Maker	*Ab Initio*	6,452	189 / 138	576 / 420	10,278 / 37	1,216,145	NA
	*Ab Initio* plus Evidence	12,645	439 / 320	1,694 / 1,251	29,663 / 66	5,548,133	27,262
Total		19,097	345 / 241	1,316/831	29,633/37	6,764, 278	27,262

Of the 19,097 predicted *D. noxia* genes and their corresponding protein models, 4,867 (25.4%) produced no BLASTP hits (E ≤ 1E^−15^) against the NCBI Insecta refseq dataset. Similarly, 4,898 *D. noxia* proteins (25.6%) were not mapped to orthologous sequences by Ortho-MCL. A BLASTN search (E ≤ 1E^−15^) of *D. noxia* transcripts *vs.* the NCBI Insecta refseq gene dataset (obtained 05/07/2014) determined that 4,867 (25.4%) *D. noxia* transcripts were unique to the species. RNAseq read mapping revealed that 2,624 (53.9%) of these unique genes were detectably expressed, while 2,243 unique genes were not (Additional file 5: Table S4). The observed percentage of distinct *D. noxia* genes is greater than that of any insect genome sequence published to date. Yet, a similar percentage of unique genes were observed in the Hessian fly *Mayetiola destructor*, a gall-forming dipteran wheat pest (personal communication, Stephen Richards). Curiously, both *M. destructor* and *D. noxia* alter wheat morphology and physiology, although through differing mechanisms, and this large percentage of unknown genes may reflect a highly evolved parasitic gene-for-gene relationship with their hosts [57,58].

### Orthology between species

Orthology analysis of the 19,097 predicted *D. noxia* proteins was performed using ORTHO-MCL on the 150-species ORTHO-MCL database. We assigned 13,402 *D. noxia* proteins (70.2%) to 7,422 ortholog groups, including 5,416 single-copy orthologs, 7,986 multi-copy orthologs, and 797 proteins that matched unassigned orthologs, for a total of 14,199 ortholog group matches. The remaining 4,898 unmatched proteins were mostly hypothetical proteins (Additional file 6: Table S5 and Additional file 7: Table S6). The majority of the 14,199 proteins matched *A. pisum* proteins more closely (81.65%), followed by other arthropods *P. humanus* (3.52%), *B. mori* (2.46%), *A. mellifera* (2.20%), *Ixodes scapularis* (1.41%), *Culex pipiens* (1.25%), *Aedes aegypti* (1.11%), *D. melanogaster* (0.88%), and *Anopheles gambiae* (0.82%) (Additional file 8: Figure S2). Primary matches to 59 additional organisms made up only 4.70% of the total known orthology designations. Among unmatched proteins, 2,649 individual paralog pairs (Additional file 9: Table S7) were identified that grouped into 357 in-paralog families containing 1,337 proteins (Additional file 10: Table S8). The three largest in-paralog families contained 35 proteins each and the smallest (207 separate groups) held two proteins each. In-paralog families were identified through comparisons to 150 separate species to ensure the greatest level of discrimination and produce the most *D. noxia*-specific in-paralog group possible.

**Figure 2:**
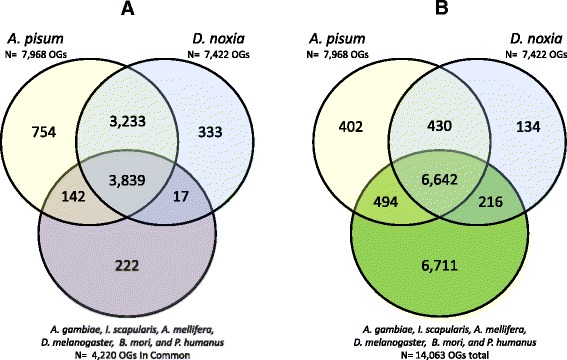
Comparison of orthology among arthropod species. **A**. Ortholog groups common to *A. gambiae*, *I. scapularis*, *A. mellifera*, *D. melanogaster*, *B. mori*, and *P. humanus* (present in all six species) in comparison to ortholog groups present in *D. noxia* and *A. pisum.* **B**. Ortholog groups present in at least one of the named species compared to ortholog groups present in *D. noxia* and *A. pisum.*

*D. noxia* and *A. pisum* share 7,072 common ortholog groups which included 2,290 single-copy genes present in both species. Ortholog groups present in *D. noxia* and *A. pisum,* when compared to other selected arthropod species (*A. gambiae, I. scapularis, A. mellifera, D. melanogaster, B. mori,* and *P. humanus*), revealed an increasing distance between aphids and other insects or arthropods (Figure 2 and Additional file 11: Figure S3). Of the 7,072 ortholog groups shared between *D. noxia* and *A. pisum*, 3,839 were common to all eight arthropods (Figure 2A). Of the remaining 3,233 OGs not common to all examined species, 430 were exclusive to *D. noxia* and *A. pisum*, and *D. noxia* possessed 134 OGs not observed in any of the other species (Figure 2B). Probing the relationship of *D. noxia* and *A. pisum* to other individual arthropod species (Additional file 11: Figure S3) found a maximum of 5,990 OGs in common with *P. humanus* and a minimum of 5,021 in common with *I. scapularis*. Evaluations of the orthological relationship between *D. noxia* and *A. pisum* and more distantly related organisms revealed fewer common ortholog groups, with a minimum of 2,378 groups in common with bread mold, *Neurospora crassa* (Additional file 11: Figure S3).

**Figure 3:**
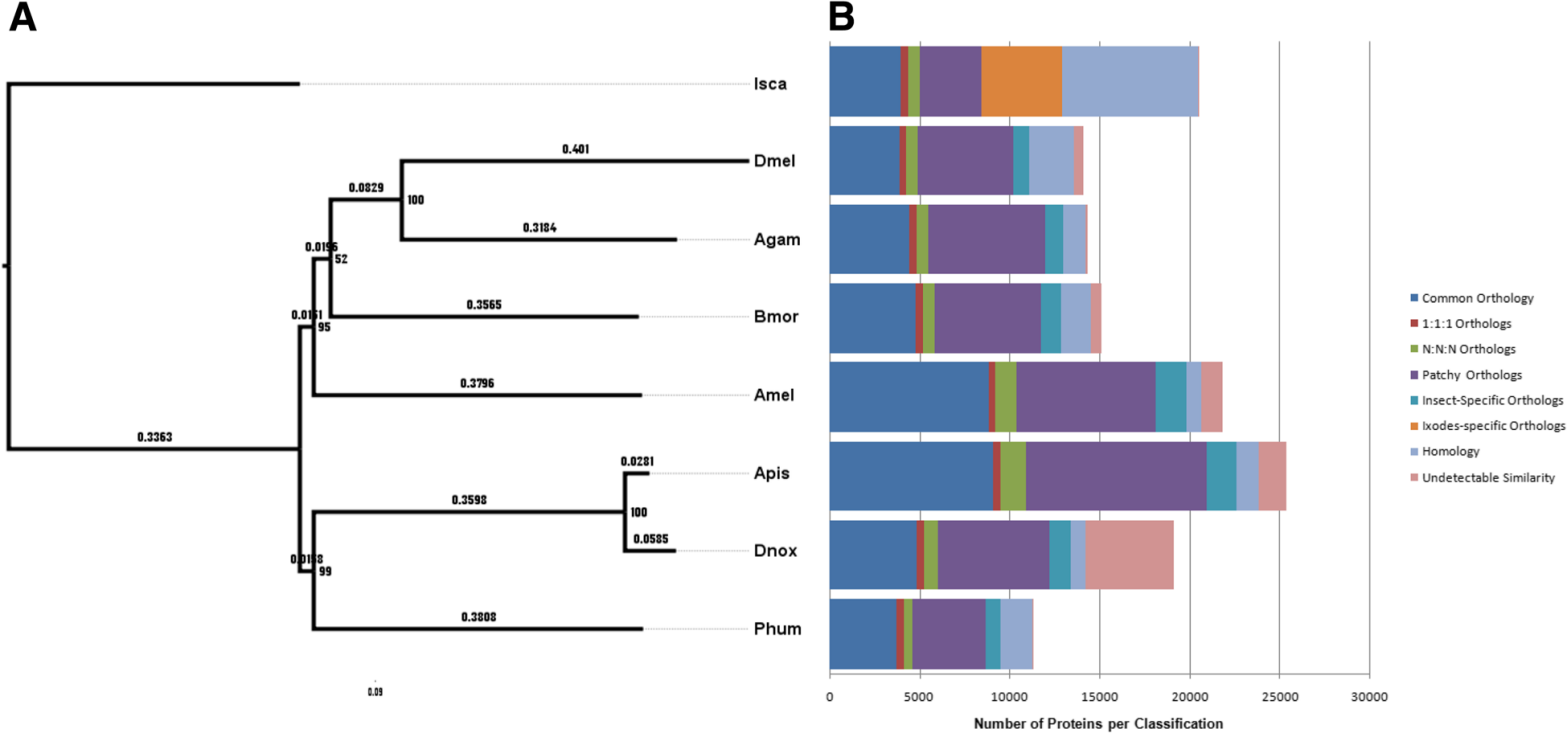
Comparison of the predicted proteomes of *D. noxia* and seven additional arthropod species. **A**. Maximum-likelihood phylogeny generated from concatenated MUSCLE alignments of each of 37 single-copy proteins unique to the listed Arthropod species. Bootstrap values (1,000 replicates) are indicated at each node. Substitutions per site are indicated on each branch. Isca, *Ixodes scapularis*, Apis, *Acyrthosiphon pisum*, Dnox, *Diuraphis noxia*, Phum, *Pediculus humanus*, Dmel, *Drosophila melanogaster*, Agam, *Anopheles gambiae*, Bmor, *Bombyx mori*, Amel, *Apis mellifera*. **B**. Comparison of gene distributions among ortholog groups: Common orthology denotes genes common to all listed species that do not follow strict 1:1:1 or N:N:N relationships among species. 1:1:1 orthologs are comprised of a single gene in all species. N:N:N orthologs are comprised of multiple genes in all species. Patchy orthologs are missing in at least one insect species. Insect-specific orthologs are present in all insect species, but absent in *I. scapularis. Ixodes*-specific orthologs are present only in *I. scapularis*. Homology denotes proteins that are assigned matches with indeterminate orthology. Undetectable similarity denotes proteins to which there is no match with an E-value < 1E^−5^ in the OrthoMCL database.

The phyletic relationship between *D. noxia* and other arthropod species [10,28,34,56,59,60] was examined by constructing a maximum-likelihood phylogeny from concatenated alignments of 37 single-copy proteins unique to arthropods (Figure 3A). Results confirmed those of previous insect phylogenetic analyses [2,10,11,33,53,55] that demonstrate an ancient branch point between insects and arachnids and an early divergence between paraneopteran insects represented by the hemimetabolic insects *D. noxia*, *A. pisum*, and *P. humanus*, and the remaining holometabolic insects. Furthermore the accurate placement of this aphid in the phylogeny of other insect groups validates the robustness of the *D. noxia* genome assembly and gene predictions.

Direct examination of orthological relationships between each species (Figure 3B) determined that of the common 3,839 OGs, 401 OGs were present in 1:1:1 relationships and 145 OGs had N:N:N relationships in all examined species, allowing no gene losses within individual species. The remaining 3,293 OGs were present in either single or multiple copies in each species, and were classified as common orthologs. Ortholog groups with losses among species, including species-specific OGs, were classified as patchy orthologs which includes 752 ortholog groups unique to insects with varying numbers of members in each species, while 2,011 OGs (4,454 proteins) were present only in *I. scapularis*. The remaining proteins for each species were classified either as homologous proteins not yet placed into orthologous groups, or as unclassified proteins with no acceptable match in the orthology database. The pattern of orthology classification in *D. noxia* is similar to other insect species, yet with a larger percentage of unclassified genes [10,28,31,34,53,56,60,61]. By disallowing orthology group losses we present the most strict representation of orthologous relationships.

### Lineage-specific expansions

**Figure 4:**
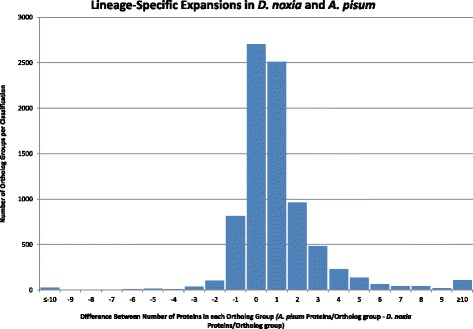
Lineage-specific expansions of ortholog groups between *D. noxia* and *A. pisum*, including ortholog groups unique to each species. The number of proteins contained within each ortholog group in *A. pisum* was subtracted from the number of proteins in the identical ortholog group in *D. noxia*. Negative numbers indicate lineage-specific expansions in *D. noxia*, and positive numbers indicate lineage-specific expansions in *A. pisum*.

Lineage-specific expansions (LSEs), reductions, and deletions for *D. noxia versus A. pisum* were analyzed by comparing Ortho-MCL analyses of their predicted proteomes. A previous LSE comparison of *A. pisum* with *P. humanus* revealed a large number of aphid specific expansions [10], and genomic expansions correspond with host race evolution in *A. pisum* [62,63]. Comparisons of gene copies per ortholog group between *D. noxia* and *A. pisum* found that most common ortholog groups contained identical gene numbers in each species. However, *A. pisum* possessed a larger number of expanded gene families (Figure 4, Additional file 12: Table S9, Additional file 13: Table S10, and Additional file 14: Table S11). *D. noxia* exhibited 1,022 lineage-specific ortholog group expansions, including 672 expanded groups (1,777 additional genes) and 350 novel groups not present in *A. pisum. A. pisum* had 4,591 ortholog group expansions, including 3,694 expanded groups (9,835 additional genes) and 895 ortholog groups not present in *D. noxia*. A total of 3,004 ortholog groups (3,261 individual genes) had equal numbers of members in *D. noxia* and *A. pisum*, including 2,290 1:1 orthologs and 413 N:N orthologs (Figure 4). Four of the five largest RWA-specific expansions were in ortholog groups associated with transposable and retrotransposable elements and an unclassified gene family, a pattern also noted in *A. pisum* [10], while the fifth largest expansion occurred in a zinc finger-associated ortholog group (50 additional genes) (Additional file 12: Table S9 and Additional file 13: Table S10). Additional large *D. noxia* ortholog group expansions included FTsJ-like methyltransferase (34 additional genes), zinc-finger proteins (78 additional genes in three groups), and alcohol dehydrogenase transcription factors (27 additional genes in three groups). In contrast, the five largest pea aphid lineage-specific expansions were Kelch proteins (286 additional genes), a retrotransposon peptidase (183 additional genes), two unclassified gene families (92 and 89 additional genes), and a zinc finger protein (79 additional genes).

*A. pisum* is thought to have undergone extensive gene duplication during its evolution [10], which our LSE comparisons with *D. noxia* affirm. The general decrease in duplications per ortholog group, and the lower abundance of ortholog groups, in *D. noxia versus A. pisum* suggests that the *D. noxia* genome has been subject to relatively less alteration over the course of its evolution. *D. noxia*’s relative lack of gene duplications and expansions may indicate that *D. noxia* maintains and increases it host range by means other than genomic alteration or gene family expansion [47,48,62,63].

### Feeding-related genes

Aphid feeding requires a balance of specific salivary components to suppress or mitigate plant defenses throughout the stylet probing and feeding processes to allow sustained feeding on host plant phloem [64,65]. The invasive nature of plant feeding by aphids requires the expression of an array of salivary and metabolic genes that act upon the plant and protect the aphid from plant defensive proteins and xenobiotics [3,64-69]. *D. noxia* is unique among most aphids in that the saliva it injects while feeding produces phytotoxic symptoms that alter plant morphology and progressively damage the host to enrich phloem nutrition [14,69-71]. In accordance with the differences in host range between aphid species, feeding-related genes would certainly be subject to variation among and within species, therefore, salivary protein profiles are distinct to aphid species, biotypes, and host races [69,72-76].

### Salivary genes

We discovered 29 of 34 salivary genes previously detected in proteomic analyses of four *D. noxia* biotypes in this genome assembly [69]. Five genes that were not detected were the *D. noxia* orthologs of GJ23220, IscW_ISCW012834, IP06594, Lava Lamp, and mitochondrial cytochrome c oxidase subunit I (COI). However, the mitochondrial COI gene was noted among the RNAseq-predicted transcripts, but was excluded from the genome assembly by the high-molecular weight DNA extraction method utilized. The remaining absent proteins may represent unassembled portions of the *D. noxia* genome, or may have sequences that are significantly altered outside of the original identified peptides [69].

A BLASTP examination comparing each predicted *D. noxia* salivary protein sequence to the NCBI Insecta refseq protein database revealed that each *D. noxia* salivary protein was more closely related to an *A. pisum* counterpart than to proteins from any other species, with E values ranging from 0.00 to 6.22E^−74^ and identities ranging from 100% to 58.21% (Additional file 15: Table S12 and Additional file 16: Table S13). The level of homology between *D. noxia* salivary protein sequences and their corresponding *A. pisum* orthologs varied inversely with the apparent abundance of each protein in the saliva [69]. Common insect salivary proteins such as glucose dehydrogenase, trehalase, and apolipophorin were among the proteins with the least homology to their *A. pisum* orthologs. In contrast, those *D. noxia* salivary proteins that have not been observed in the saliva of other insects exhibited greater homology with orthologs from *A. pisum* and other insect species (Additional file 15: Table S12 and Additional file 16: Table S13) [69,73]. This finding implies that salivary gene expression, rather than sequence divergence, may play a role in *D. noxia*’s host specificity and phytotoxicity.

Glucose dehydrogenase and apolipophorin are among the most common and abundant proteins in aphid saliva [66,69,73,74]. Multiple glucose dehydrogenase proteins are present in aphid saliva, but their differing amino acid compositions suggest that each protein performs a different function within the plant host. Apolipophorin, present as a single gene copy in *D. noxia*, *A. pisum*, and most other insect species, was used to examine the phylogenetic relationship of *D. noxia* with other arthropods from the perspective of a conserved single-copy gene. A maximum-likelihood phylogenetic tree derived from a MUSCLE alignment of apolipophorin from eleven arthropod species confirmed known phylogenetic patterns, with basal branching of the aphid lineage from the holometabola and a more recent divergence of *D. noxia* and *A. pisum* (Additional file 17: Figure S4).

### Defensive and detoxifying genes

**Figure 5:**
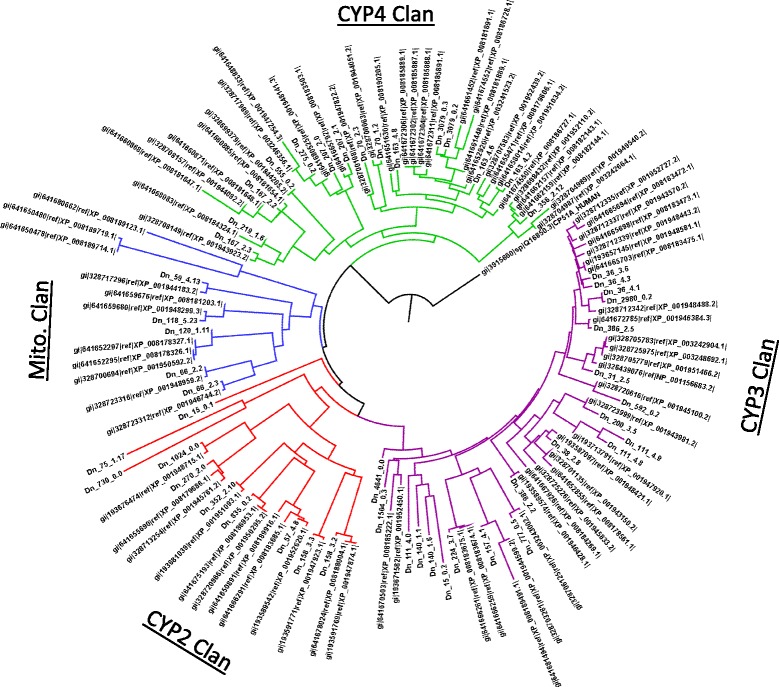
Maximum-likelihood phylogeny of cytochrome P450 genes in *D. noxia* and *A. pisum*. Cytochrome P450s of both *D. noxia* and *A. pisum* were aligned using CLUSTAL-W and then subject to a maximum-likelihood phylogeny using CLC genomics workbench. CYP450s from each species cluster into like groups of CYP450 clans 2, 3, 4, and mitochondrial. Human CYP51A was used as a rooting outgroup.

Insects possess a suite of defensive and detoxification genes in order to cope with constitutive and induced host defensive compounds and xenobiotics, [65-68]. The most important insect defense and detoxification genes include ABC transporters (ABCt), cytochrome P450s (CYP450), glutathione-s transferases (GST), and carboxyl and choline esterases (CCE) [30,33,53,77]. *D. noxia* possesses 53 ABCt, 48 CYP450, 11 GST, and 8 CCE genes, compared to 113 ABCt, 85 CYP450, 28 GST, and 29 CCE genes in *A. pisum* (Additional file 1: Table S1, Additional file 12: Table S9, and Additional file 16: Table S13). We performed a phylogenetic analysis of CYP450 protein sequences from *D. noxia* and *A. pisum* in order to examine the relationship between the two species. CYP450 proteins from each species, representing CYP clans 2, 3, and 4, as well as the mitochondrial CYP clan, grouped together, validating the accuracy of the assembly and annotations, as well as demonstrating an evolutionarily close relationship between the two species (Figure 5). The close relationship between *D. noxia* and *A. pisum* is further demonstrated by the 89.2% median similarity between CYP450s from the two species. Although five *D. noxia* CYP450s belonged to the mitochondrial clan, the *D. noxia* mitochondrial genome contains no CYP450 sequences [78], nor did BLASTP analysis reveal the presence of any *D. noxia* mitochondrial proteins in this assembly. Thus each mitochondrial CYP450 sequence noted in the *D. noxia* genome may represent an instance of horizontal gene transfer during the early evolution of its primordial aphid ancestor. GSTs had a median 92.1% identity between the two aphid species, and CCEs had a median 91.2% identity between the two aphid species. The reduced number of defensive and detoxification proteins for *D. noxia* may reflect a greater role of phytotoxic salivary effects and decreased reliance upon physiological and metabolic countermeasures to host defenses in comparison with *A. pisum* and other insects in general.

### Chemoreceptors

Chemoreception genes are critical in perceiving taste and odor stimuli in order to locate appropriate food sources and establish feeding. Duplication or mutation of chemoreceptor genes can alter feeding behavior, and is implicated in insect speciation [48,62,63] and in establishing host range [79]. The *D. noxia* genome contains 30 gustatory receptors (GR), 21 odorant receptors (OR), and 9 odorant binding proteins (OBP) (Additional file 16: Table S13), while *A. pisum* has 77 GRs, 79 ORs, and 15 OBPs [62] and *Aphis gossypii*, a generalist feeder, has 45 ORs, but an unreported GR and OBP number [80]. Another Hemipteran insect, *P. humanus*, has only 10 ORs, 5 OBPs, and 8 GRs, a condition suspected to result from host range restriction [34]. Omnivorous insect species also have a much higher number of chemoreceptors; the omnivorous *T. castaneum* possesses 265 ORs and 220 GRs [33], the housefly *Musca domestica* has 52 OBPs, 62 ORs, and 68 GRs [81], and the hymenopteran nectar-feeder *A. mellifera* has 170 ORs and 21 OBPs, but only 10 GRs [28]. Comparison of OR numbers across insect species is complicated by the fact they include receptors to detect sexual pheromones that are essential to reproduction. Accordingly, high sequence variability was found between the ORs of *D. noxia* and *A. pisum*, ranging from 95% to 28% identity with the corresponding *A. pisum* OR sequence. Substantial sequence variation was also noted between *A. gossypii* and *A. pisum* ORs [80], indicating their potential role in host selection. The scarcity of *D. noxia* chemoreceptors in comparison with *A. pisum* and *A. gossypii* suggests that taste and odor perception may be less important in food source selection for *D. noxia*. Reductions in chemoreceptor numbers suggests that *D. noxia* relies upon phytotoxic salivary proteins to overcome host defenses and enhance the nutritional value of its hosts, thereby reducing its reliance upon chemoreceptors to identify suitable hosts and to broaden its host range [70,71].

### Sugar transporters

Aphids consume a sugar-rich diet with a high osmotic potential, requiring only proteins such as uniporters that allow movement of phloem sugars with the membrane concentration gradient and into the hemolymph [10]. The *D. noxia* genome contains a number of sugar transporters, including 84 Major facilitator genes compared with 200 in *A. pisum* and 13 inositol/glucose/sugar transporters *versus* 34 in pea aphid [10] (Additional file 16: Table S13). It is hypothesized that the relative increase in *A. pisum* sugar transporters in comparison to other sequenced insects reflects the adaptation to a sugar-rich diet [10]. *D. noxia* has a lower number of sugar transporters *D. noxia* relative to *A. pisum*, revealing that sugar transporter gene expansion is not a universal condition in aphids and varies by hosts they utilize.

### RNAi and epigenetic pathways

The RNA regulatory pathway, which includes the RNA interference (RNAi) and epigenetic regulatory pathways, functions in viral defense and gene regulation by degrading aberrant RNA and establishing and maintaining DNA and chromatin methylation. These mechanisms are not present in all insect lineages [41,42,82], and are notably lacking in *D. melanogaster* [38]. Regulation of gene expression by DNA methylation is an essential aspect of polyphenism in aphids and other insects [41,42]. Likewise, *D. noxia* possesses the components of the common insect RNAi and epigenetic pathways [41,82-84]. Single copies of the genes SID1, AGO3, DCR-1, DCR-2, Drosha, Pasha, vacuolar H + −ATPase, Exportin-5, HEN1, Loquacious, and R2D2 were found, along with five PIWI, two PRMT-5, two AGO1, and two AGO2 genes (Additional file 16: Table S13). Genes required for epigenetic DNA and chromatin modifications were also present, including six Type 1 and 3 DNA methyltransferases, 16 histone-lysine methyltransferases, and 10 histone deacetylases (Additional file 16: Table S13). The presence of RNAi, DNA methylation, and chromatin methylation pathway components in *D. noxia*, in conjunction with the existence of a bimodal CpG_(O/E)_ distribution ratio (Figure 1), confirms that *D. noxia* genes are subject to regulatory methylation similar to *A. pisum* and *A. mellifera* [38,43].

### Insecticide resistance pathways

Most insecticides target specific protein motifs, and lose efficacy when mutations or alternate isoforms of the target protein prevail throughout a pest population. *D. noxia* is resistant to many insecticides in comparison to other insects [85], but is effectively controlled by systemically-applied pyrethroid, organophosphate, and organochlorine insecticides [86]. The emergence of new *D. noxia* insecticide resistance has not been reported, but the aphids *Myzus persicae*, *Aphis gossypii,* and *Schizaphis graminum* have each developed resistance to several previously-effective insecticides [87-89].

*D. noxia* possesses common insecticide targets including an acetylcholinesterase-1 ortholog with S431 pirimicarb susceptibility, four additional acetylcholinesterases, 21 acetylcholine receptors, 12 sodium channel genes, and five GABA receptors, but neither neonicotinoid-detoxifying CYP450 (CYP2A6 and CYP6CY3) [88] (Additional file 15: Table S12). The absence of reported insecticide resistance in *D. noxia* is likely due to past reliance upon host resistance instead of insecticides. However, *D. noxia* displays significant chromosomal heterogeneity and rapid biotype development under the selection pressure of plant resistance genes, making it likely that genetically-based insecticide resistance can occur under high selection pressure. *D. noxia*’s smaller complement of detoxifying genes in comparison with other insects, exemplified by the absence of CYP2A6 and CYP6CY3, further suggests that such resistance will most likely occur as a result of a mutation-based sequence shift [90], rather than through amplified expression of a rare transcript [87], although both mechanisms are possible.

### Virus transmission

The majority of aphid-related plant damage is through plant virus transmission during feeding, and most grain aphid species are significant vectors of the barley yellow dwarf virus [91]. *D. noxia* is exceptional in that it does not transmit plant pathogenic viruses [18]. Nevertheless, the genome of *D. noxia* possesses a full complement of proteins thought to be involved in viral transfer, including 10 dynamins, 8 serine protease inhibitors, 8 vesicle transport/trafficking proteins, and 15 cyclophilins [10,15] (Additional file 16: Table S13). As viruses interact with specific epitopes of proteins involved in trans-membrane transport, it is likely that protein sequence differences between *D. noxia* and virus-transmitting aphids do not favor viral attachment. The inability of *D. noxia* to vector viruses requires further exploration.

### Genes laterally transferred from bacteria

Aphids are obligate parasites that are able to feed upon nutritionally-deficient phloem sap through an endosymbiotic relationship with *Buchnera aphidicola*. These bacteria are housed within specialized bacteriocytes in the aphid gut lining and produce essential amino acids lacking in the host plant phloem [92]. *B. aphidicola* displays limited sequence and gene copy number variance between *D. noxia* biotypes, and it is hypothesized that variance in total endosymbiont and plasmid copy number impacts aphid fitness [92,93]. The *D. noxia* genome holds genes that originated from the genome of *B. aphidicola* and that represent horizontal gene transfer from the *B. aphidicola* genome to the *D. noxia* genome. These include one LD carboxypeptidase and one rare lipoprotein receptor (RlpA) (Additional file 16: Table S13) as found in *A. pisum* [10,94,95], but not the acetylmuramidases noted in *A. pisum* [10]. These genes were each located within long contigs (>5,000 bases in length) that included additional *D. noxia* genes not derived from the endosymbiont. As in *A. pisum*, there is no evidence of extensive horizontal gene transfer in the *D. noxia* genome [10]. The DNA extraction and *D. noxia* pre-assembly read filtering method removed reads matching the *B. aphidicola* assembly originating from *A. pisum,* thereby eliminating the endosymbiont genome from our analysis, as supported by the absence of mitochondrial sequence in this assembly, and thus it is not addressed.

## Conclusions

*D. noxia*’s genome shares many genes in common with the current model aphid, *A. pisum,* but varies in genome size and architecture, and specific functional genetic processes. The *D. noxia* genome, with its moderate transposable and repetitive element component and fewer total genes and gene families than are present in *A. pisum* [10], presents a case for a high degree of genomic conservation over time. The reduced repetitive element percentage in the *D. noxia* genome may factor in the lower number of gene family expansions relative to *A. pisum* [55], and is consistent with the hypothesis that insect evolution is driven by transposable element expansion and gene duplication [10,53,55,63]. The *D. noxia* genome also differs from that of *A. pisum*, primarily in genes governing host detection, acceptance, and feeding processes. This genome assembly describes *D. noxia* as a species uniquely adapted to feed upon graminaceous hosts using its salivary proteins to alter host morphology and metabolism [69-71], and provides an important contrast to non-phytotoxic aphids that depend on metabolically countering plant defensive compounds [3,66,67].

*D. noxia* possesses a low number of chemoreceptor genes compared to other insects [10,53,55,60,80] suggesting it has a low reliance on taste and odor perception as a survival criterion. It also has significantly fewer detoxifying and defensive genes in comparison with *A. pisum* and other insects [10,33,81], implying that *D. noxia* has evolved another way to circumvent host defenses. *D. noxia*’s relatively wide host range and rapid establishment into new geographical areas indicates that *D. noxia*’s genomic deficiencies in feeding-related genes in comparison to *A. pisum* are compensated for, and overcome by, phytotoxic salivary proteins that drive phloem nutrition enrichment and alter host morphology [14,69-71]. Aphids causing phytotoxic reactions in plants are uncommon, thus *D. noxia* is an exception to the typical view of insect-plant coevolution, in which aphid evolution is thought to be driven by the necessity to avoid or detoxify newly-evolving plant defensive responses in order to feed without damaging the host [96,97]. *D. noxia* presents a more rapacious character, surviving by inducing phytotoxic symptoms which damage and eventually destroy its host.

Our assembly presents a phytotoxic aphid model as an alternative genomic model for aphids and represents the second sequenced aphid genome. The contrasting and divergent evolutionary paths of *D. noxia* and *A. pisum*, and their contrasting aphid-host relationships, provide an extraordinary opportunity to better address the genetic basis of the feeding processes of aphids and their ability to evade plant defenses, to understand the nature of interactions between aphid virulence genes and plant resistance genes, and to formulate comparative and functional genomics studies that will ultimately lead to increased knowledge of aphid biology and evolution.

## Methods

### DNA and RNA collection, sequencing, and assembly

Chromosomal DNA was collected using the Agilent DNA extraction kit from a pooled sample of 200 *Diuraphis noxia* Biotype 2 adult females isolated from a single clone-derived colony obtained from the USDA-ARS Cereal Insects Genetic Resource Library (CIGRL, Stillwater, OK) reared on wheat cv. TAM110. Total RNA was also recovered from 200 pooled RWA2 adult females from the same source, and extracted using the Promega SV Total RNA Isolation system. Recovered DNA and RNA was frozen at −80°C immediately and used in subsequent sequencing analyses. The recovered DNA was sheared into paired-end and mated-pair libraries (Corvaris S2, Paired-end: peak power 50.0, duty factor 10.0, cycle per burst 200, time per run 90 s; Mated-pair: duty cycles 20%, intensity 0.1, cycle per burst 100, time per run 5 min), and purified (Paired-end: Dynal magnetic M 280-streptavidin beads, Mated-pair: Agencourt AMPure XP beads). Paired-end reads were then end-repaired, A-tailed, and ligated to adapters, then amplified by PCR (98°C for 30s, 18 cycles of: 98°C 10s, 65°C 30 s, 72°C 30s, with a final step of 72°C 15 m and 4°C until retrieved). Agencourt AMPure XP beads were used for purification following PCR. Sequencing was performed with an Illumina Hiseq 2000 with TruSeq v3.0 chemistry. Paired-end fragments, prepared by the U.S. National Institutes of Health/National Cancer Institute, averaged 223 bases with a read length of 2×101 bases. A mated-pair library prepared by the NIH/NCI averaged 2.6 kb in length, also with a read length of 2x101 bases. An additional mated-pair library was created by Axeq Technologies, Inc. (Rockville, MD) averaging 8.7 kb, with a read length of 2×101 bases. All reads were quality filtered on the basis of each read containing a minimum of 90% of bases in each read having a minimum quality score of Q20. Reads were additionally filtered before assembly by removing those reads mapping to the *A. pisum* endosymbiont *Buchnera aphidicola* genome. The quality- and *Buchnera*-filtered reads were then used as input for the genome assembly program AllPaths-LG [98,99], which was used to conduct a de novo assembly of the RWA2 genome using default settings, with inward-oriented paired-end libraries and outward-oriented jumping libraries, and with ploidy set to 2 (diploid).

RNA-seq was performed by NIH/NCI, 1 μg of RWA2 RNA per lane was processed according to the Illumina Truseq RNA Low-sample preparation protocol and sequenced using paired-end reads (2×101) on an Illumina Hiseq 2000 using Truseq v 3.0 chemistry. Reads were quality-filtered prior to assembly to include only sequences with a Q20 value in greater than 90% of bases, and these reads were used to perform a de novo transcriptome assembly using the TRINITY (r2012_10_05) software package using default settings (Broad Institute, Boston, MA) [100]. The assembled sequences were used downstream for evidence during genome annotation, and RNAseq reads were mapped to predicted transcripts using CLC genomics workbench v. 7.5.

### Transposable and repetitive element analysis

The RWA genome scaffolds were used to determine the repeat content of the RWA2 genome by analysis with RepeatMasker 4.0.3 [101]. The RWA scaffold file was analyzed using first RepeatModeler [102] to identify RWA-specific repeats. Masked sequences were then analyzed with RepeatMasker, run with the RepBase full repeat database (Repbase18.07) as an evidence file, to identify all repeats and transposable elements within the *D. noxia* genome.

### Structural prediction and genome annotation

Structural genome annotation was performed by utilizing RWA2 genomic scaffolds as input for the MAKER [103] genome annotation pipeline. RepeatMasker was used to mask low-complexity regions and repetitive DNA using the custom database created during repeat masking [101]. The following evidence files were used to aid in annotation: EST/RNA sequence evidence was provided by RWA2 Trinity-assembled RNA seq data, repetitive sequences were provided by the combined *D. noxia*/RepBase repeat database and protein data was provided by the *A. pisum* refseq protein dataset (NCBI refseq, downloaded 03/15/14). Augustus [104] was used within the MAKER framework to develop *ab initio* protein and transcript predictions. PFAM analysis was conducted using an HMM-based search (CLC Genomics version 7.0) of all MAKER-derived protein models using the full PFAM database (version 22.0). Transcripts and proteins predicted by MAKER were subjected to BLASTN and BLASTP comparisons using the CLC Genomics workbench (v. 7.0).

### Genomic analyses

Ortho-MCL [105] was used to determine the orthology of the 19,097 MAKER-identified RWA2 proteins and the NCBI protein refseq databases for *D. melanogaster* (14,067), *A. pisum* (24,378), *A. mellifera* (21,780), *P. humanus* (11,336), *A. gambiae* (14,341), *B. mori* (15,068), and *I. scapularis* (20,467) as comparison species. Orthologous groups were determined utilizing the Ortho-MCL web service (orthomcl.org). First, an all-vs-all BLASTP of each species-specific database was performed against the full OrthoMCL database (150 species, accessed 07/15/2014), followed by determination of orthologs, paralog pairs, and in-paralog groups. Results from each of these analyses were compared directly to discover multiple- and single-copy orthologs between species. In order to compare single-copy orthologs between species, 37 single-copy orthologs specific to this arthropod group, and absent from any other organism, were retrieved from the ORTHO-MCL database and aligned using MUSCLE [106]. The resulting alignments were concatenated by CLC genomics workbench (v. 7.0). Concatenated alignments were used to construct a maximum-likelihood phylogeny by neighbor-joining analysis over 1,000 replicates, also using the CLC genomics workbench (v. 7.0). Additional phylogenetic analyses were conducted using MUSCLE or CLUSTAL-W alignments to produce maximum-likelihood phylogenies by neighbor-joining analysis with the CLC genomics workbench (v. 7.0)

Nucleotide and dinucleotide content of the genome and predicted transcripts was conducted using Sequool software package. Percentages of each nucleotide per scaffold or transcript were analyzed, as were the percentage of CpG dinucleotides. CpG dinucleotide observed/expected ratio was performed for each transcript using the formula CpG_(O/E)_ = CpG frequency/(C frequency × G frequency) [43].

### Data access

The Whole Genome shotgun project was deposited with the National Center for Biotechnological Information (NCBI) under accession number JOTR00000000, Bioproject PRJNA233413. Raw Illumina DNA reads were submitted to the NCBI SRA database under the Biosample number SAMN02693874, RNAseq reads were submitted under biosample number SAMN03435929. Illumina reads may be accessed under SRA study SRP040557.

## Additional files

Additional file 1: Table S1. Expression of individual genes in FPKM as measured by mapping of RNAseq reads against predicted *D. noxia* gene sequences. Additional file 2: Table S2. CpG frequencies and observed/expected ratios among predicted *D. noxia* transcripts. Additional file 3: Figure S1. Analysis of genome size *versus* repetitive element content of selected arthropods. Additional file 4: Table S3. PFAM domains identified in predicted *D. noxia* proteins. Additional file 5: Table S4. RNAseq read mapping of genes unique to *D. Noxia*. Additional file 6: Table S5. Predicted *D. Noxia* genes with orthologous matches. The orthology classifications, top orthologous matches, and number of genes per ortholog group are included. Additional file 7: Table S6. Orthology analysis of complete *D. noxia* gene set. Additional file 8: Figure S2. Distribution of top hits to *D. noxia* genes. Additional file 9:Table S7. Paralog pairs identified during orthology analysis of predicted *D. noxia* proteins. Additional file 10: Table S8. In-paralog groups identified from merged paralog pairs. Additional file 11: Figure S3. Venn diagrams describing orthology relationships between *D. noxia*, *A. pisum*, and each indicated species. Additional file 12: Table S9. Lineage-specific ortholog group expansions, reductions, and deletions between *D. noxia* and *A. pisum*. Additional file 13: Table S10. Lineage-specific ortholog group expansions and reductions between *D. noxia* and *A. pisum*. Additional file 14: Table S11. Ortholog group deletions among *D. noxia* and *A. pisum*. Additional file 15: Table S12. BLASTP analysis of previously-detected *D. noxia* salivary protein-coding genes. Additional file 16: Table S13. List of genes comprising the functional groups identified in *D. noxia* Biotype 2. Additional file 17: Figure S4. Phylogeny of apolipophorin among selected species.

## Abbreviations

G + C: Guanine + Cytosine
A + T: Adenine + Thymidine
SNP: Single nucleotide polymorphism
FISH: Fluorescence *In-Situ* Hybridization
HMM: Hidden Markov Model
CEG: Conserved Eukaryotic Gene
SINE: Short Interspersed Nuclear Element
LINE: Long Interspersed Nuclear Element
LTR: Long Terminal Repeat
RWA: Russian wheat aphid
EST: Expressed sequence tag
NCBI: National Center for Biotechnology Information
Apis: *Acyrthosiphon pisum*
Dnox: *Diuraphis noxia*
Isca: *Ixodes scapularis*
Bmor: *Bombyx mori*
Phum: *Pediculus humanus*
Amel: *Apis mellifera*
Dmel: *Drosophila melanogaster*
Agam: *Anopheles gambiae*
OG: Ortholog group
LSE: Lineage-specific expansion
ABCt: ABC transporter
CYP450: Cytochrome P450
GST: Glutathione-S tranferase
CCE: Carboxy/Choline esterase
GR: Gustatory receptor
OR: Odorant receptor
OBP: Odorant binding protein
GABA: Gamma-aminobutyric acid
