# Supplementary material for: The genome of Diuraphis noxia, a global aphid pest of small grains
Source: BMC Genomics. 2015 Jun 5;16:429. doi: 10.1186/s12864-015-1525-1 (PMC4561433; doi:10.1186/s12864-015-1525-1)
Supplement: Additional file 3: Figure S1. — Analysis of genome size versus repetitive element content of selected arthropods. [file 12864_2015_1525_MOESM3_ESM.pptx]

## Slide 1
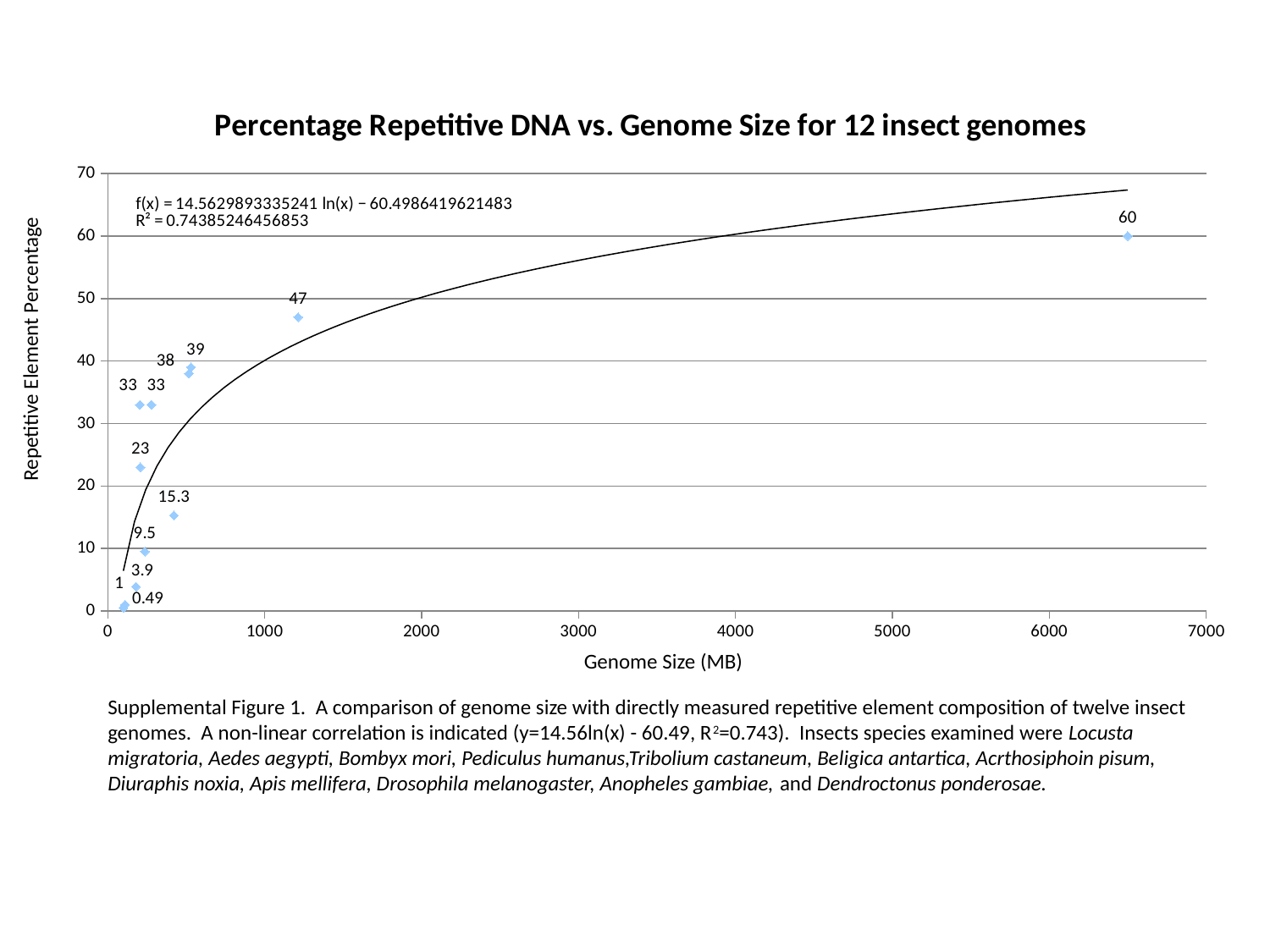

### Chart: Percentage Repetitive DNA vs. Genome Size for 12 insect genomes
| Category | repeat percentage |
|---|---|Repetitive Element Percentage
Genome Size (MB)
Supplemental Figure 1. A comparison of genome size with directly measured repetitive element composition of twelve insect genomes. A non-linear correlation is indicated (y=14.56ln(x) - 60.49, R2=0.743). Insects species examined were Locusta migratoria, Aedes aegypti, Bombyx mori, Pediculus humanus,Tribolium castaneum, Beligica antartica, Acrthosiphoin pisum, Diuraphis noxia, Apis mellifera, Drosophila melanogaster, Anopheles gambiae, and Dendroctonus ponderosae.
