# Supplementary material for: The genome of Diuraphis noxia, a global aphid pest of small grains
Source: BMC Genomics. 2015 Jun 5;16:429. doi: 10.1186/s12864-015-1525-1 (PMC4561433; doi:10.1186/s12864-015-1525-1)
Supplement: Additional file 8: Figure S2. — Distribution of top hits to D. noxia genes. [file 12864_2015_1525_MOESM8_ESM.pptx]

## Slide 1
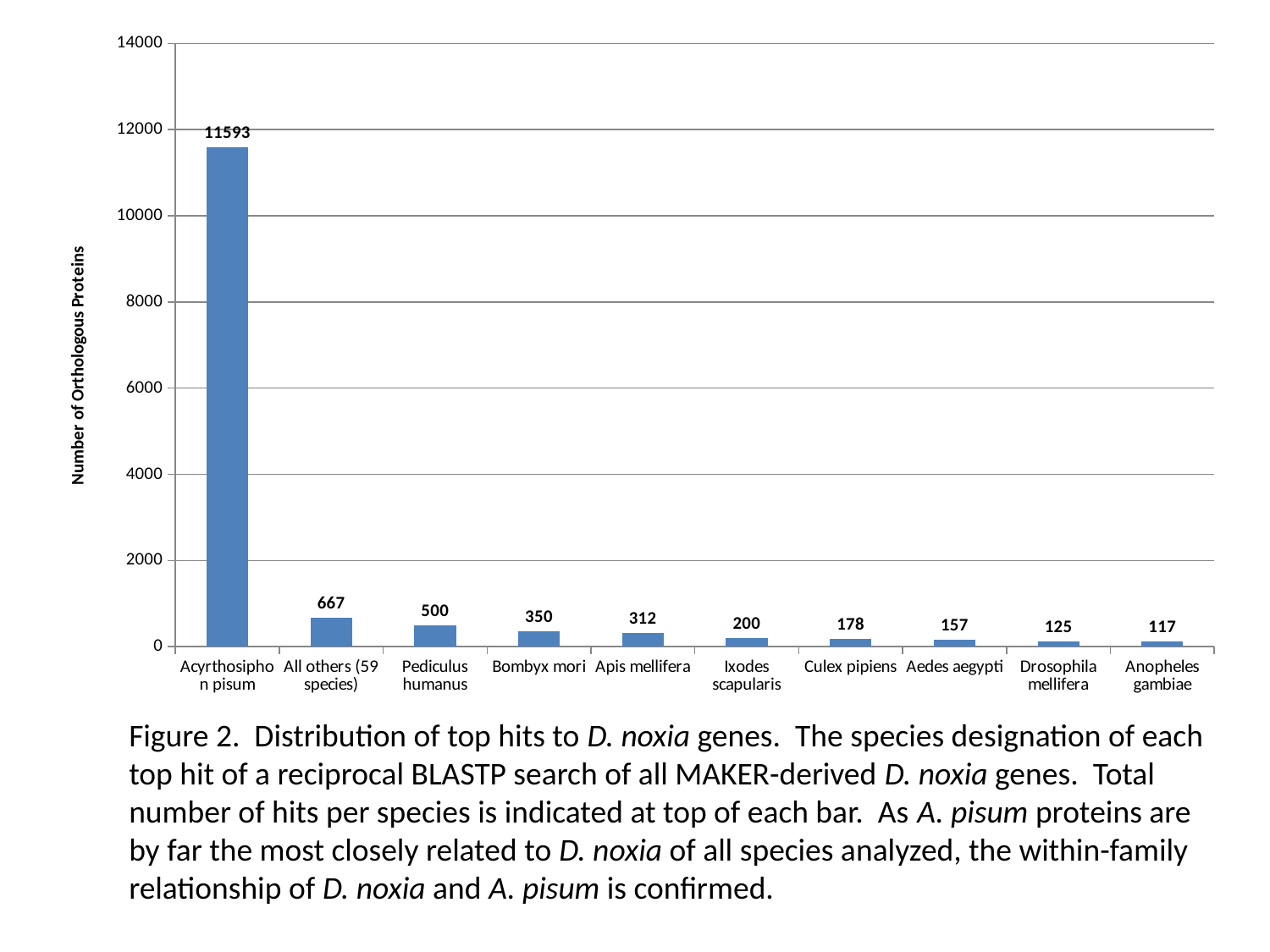

### Chart
| Category | |
|---|---|
| Acyrthosiphon pisum | 11593.0 |
| All others (59 species) | 667.0 |
| Pediculus humanus | 500.0 |
| Bombyx mori | 350.0 |
| Apis mellifera | 312.0 |
| Ixodes scapularis | 200.0 |
| Culex pipiens | 178.0 |
| Aedes aegypti | 157.0 |
| Drosophila mellifera | 125.0 |
| Anopheles gambiae | 117.0 |Figure 2. Distribution of top hits to D. noxia genes. The species designation of each top hit of a reciprocal BLASTP search of all MAKER-derived D. noxia genes. Total number of hits per species is indicated at top of each bar. As A. pisum proteins are by far the most closely related to D. noxia of all species analyzed, the within-family relationship of D. noxia and A. pisum is confirmed.
