# Supplementary material for: The genome of Diuraphis noxia, a global aphid pest of small grains
Source: BMC Genomics. 2015 Jun 5;16:429. doi: 10.1186/s12864-015-1525-1 (PMC4561433; doi:10.1186/s12864-015-1525-1)
Supplement: Additional file 11: Figure S3. — Venn diagrams describing orthology relationships between D. noxia, A. pisum, and each indicated species. [file 12864_2015_1525_MOESM11_ESM.pptx]

## Slide 1
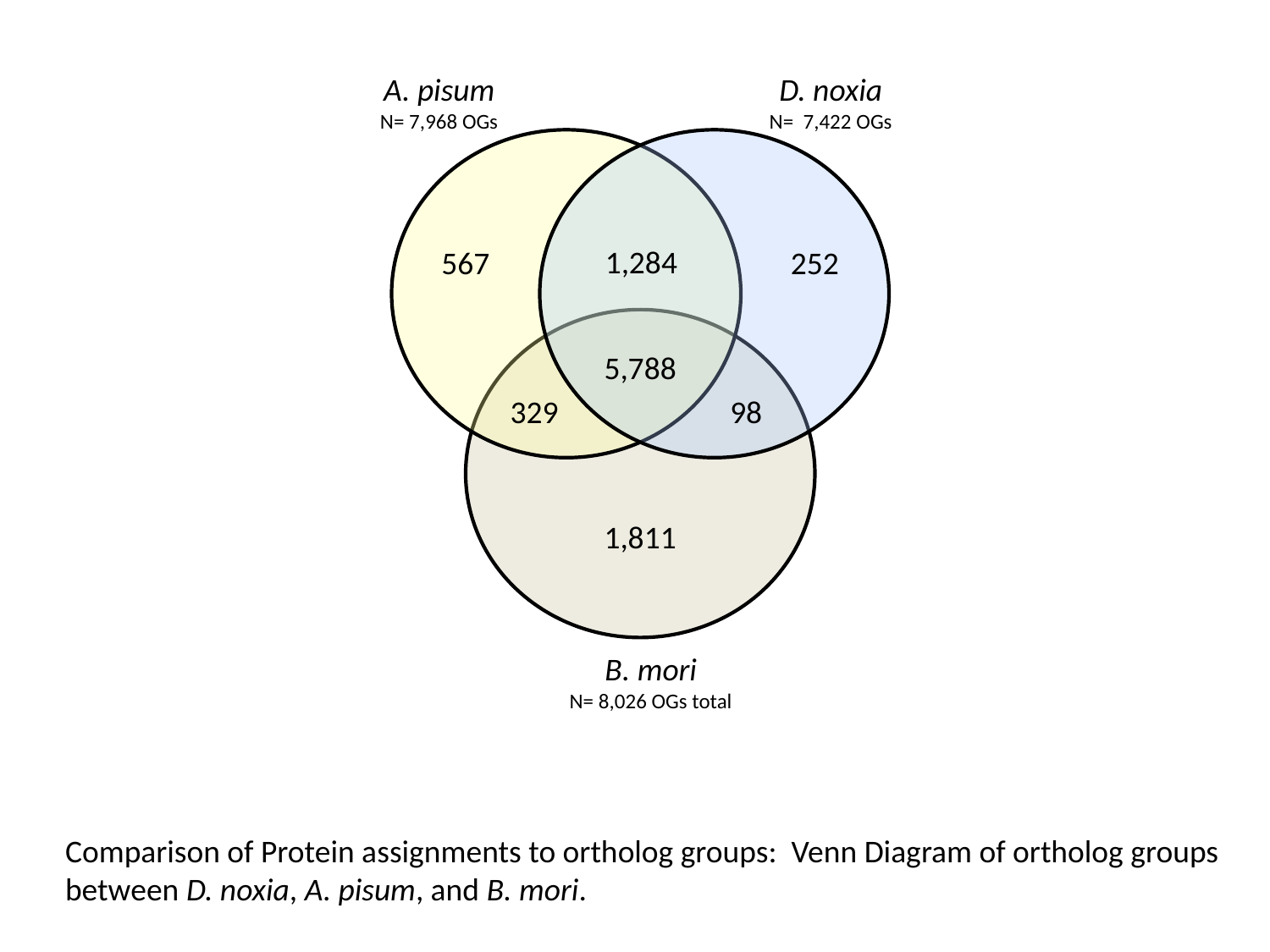

A. pisum
N= 7,968 OGs
D. noxia
N= 7,422 OGs
1,284
567
252
5,788
329
98
1,811
B. mori
N= 8,026 OGs total
Comparison of Protein assignments to ortholog groups: Venn Diagram of ortholog groups between D. noxia, A. pisum, and B. mori.

## Slide 2
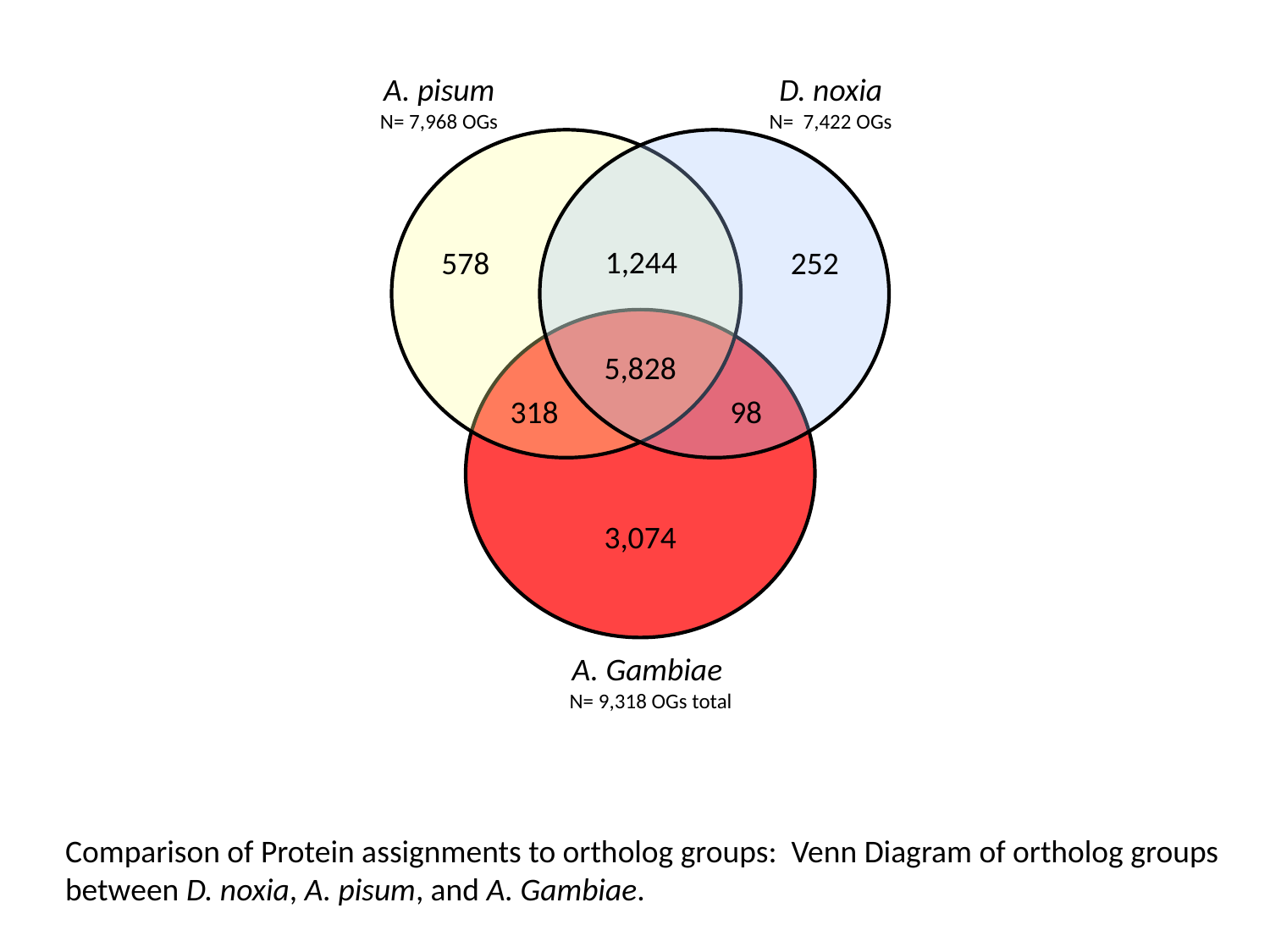

A. pisum
N= 7,968 OGs
D. noxia
N= 7,422 OGs
1,244
578
252
5,828
318
98
3,074
A. Gambiae
N= 9,318 OGs total
Comparison of Protein assignments to ortholog groups: Venn Diagram of ortholog groups between D. noxia, A. pisum, and A. Gambiae.

## Slide 3
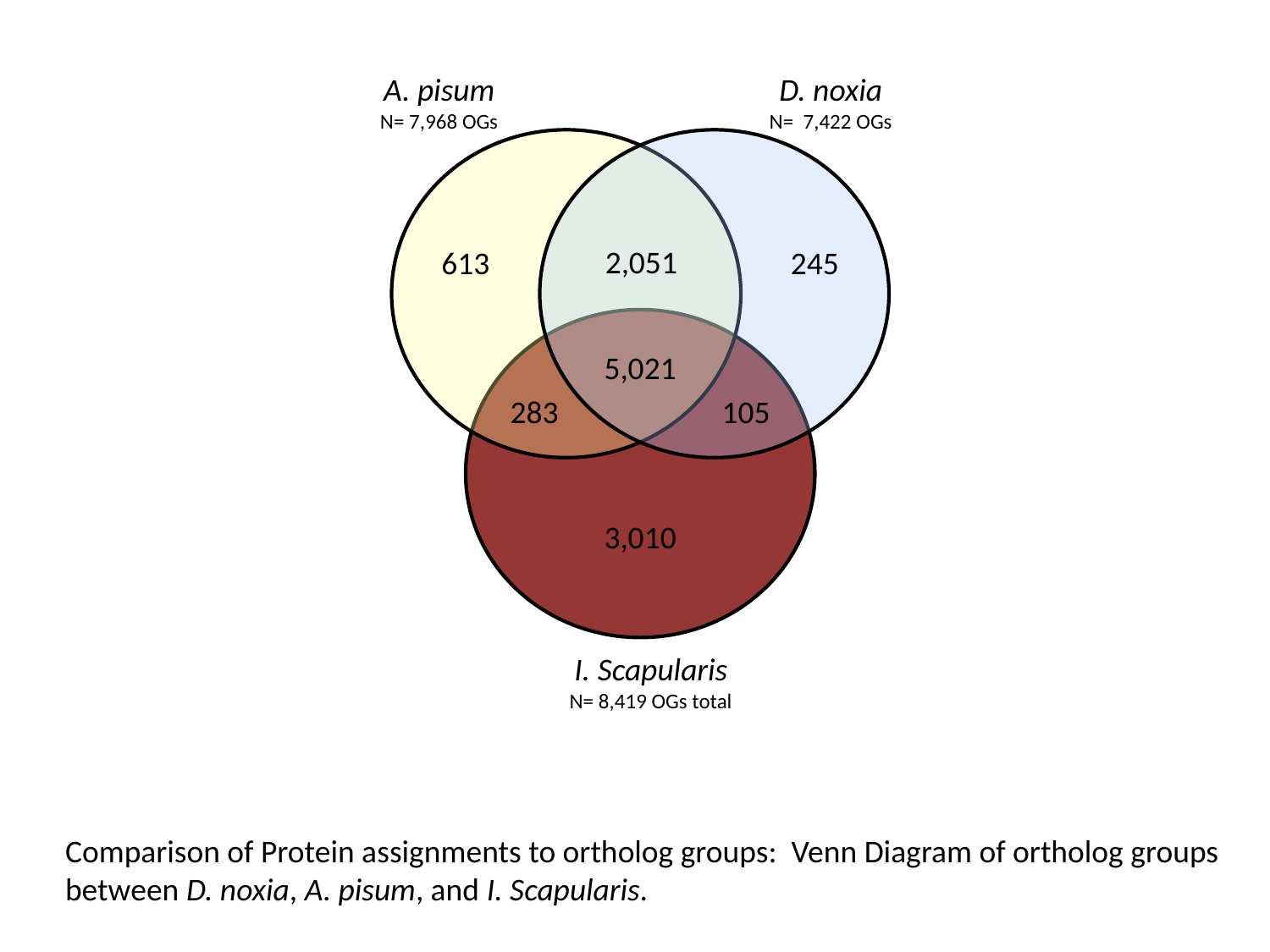

A. pisum
N= 7,968 OGs
D. noxia
N= 7,422 OGs
2,051
613
245
5,021
283
105
3,010
I. Scapularis
N= 8,419 OGs total
Comparison of Protein assignments to ortholog groups: Venn Diagram of ortholog groups between D. noxia, A. pisum, and I. Scapularis.

## Slide 4
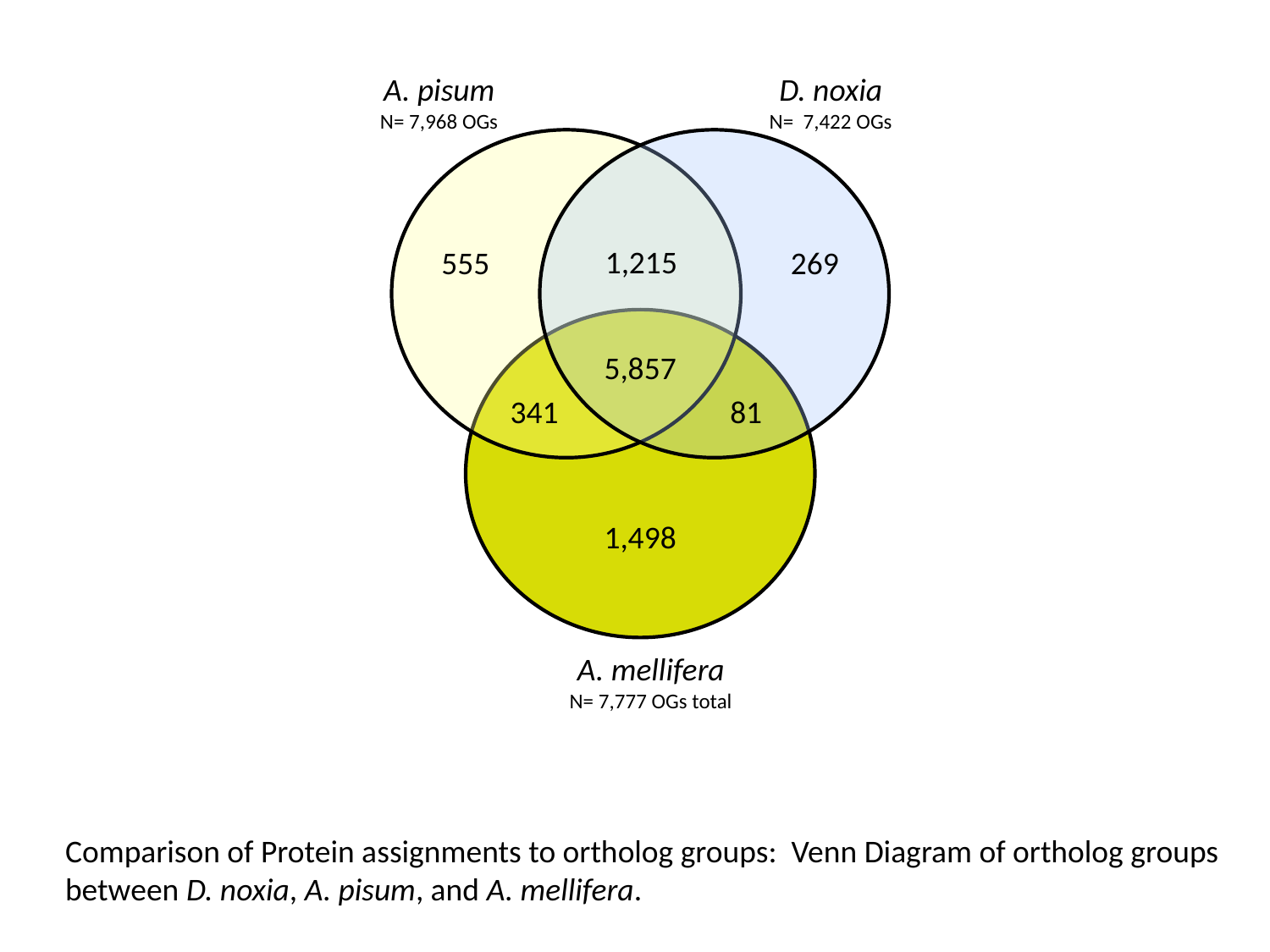

A. pisum
N= 7,968 OGs
D. noxia
N= 7,422 OGs
1,215
555
269
5,857
341
81
1,498
A. mellifera
N= 7,777 OGs total
Comparison of Protein assignments to ortholog groups: Venn Diagram of ortholog groups between D. noxia, A. pisum, and A. mellifera.

## Slide 5
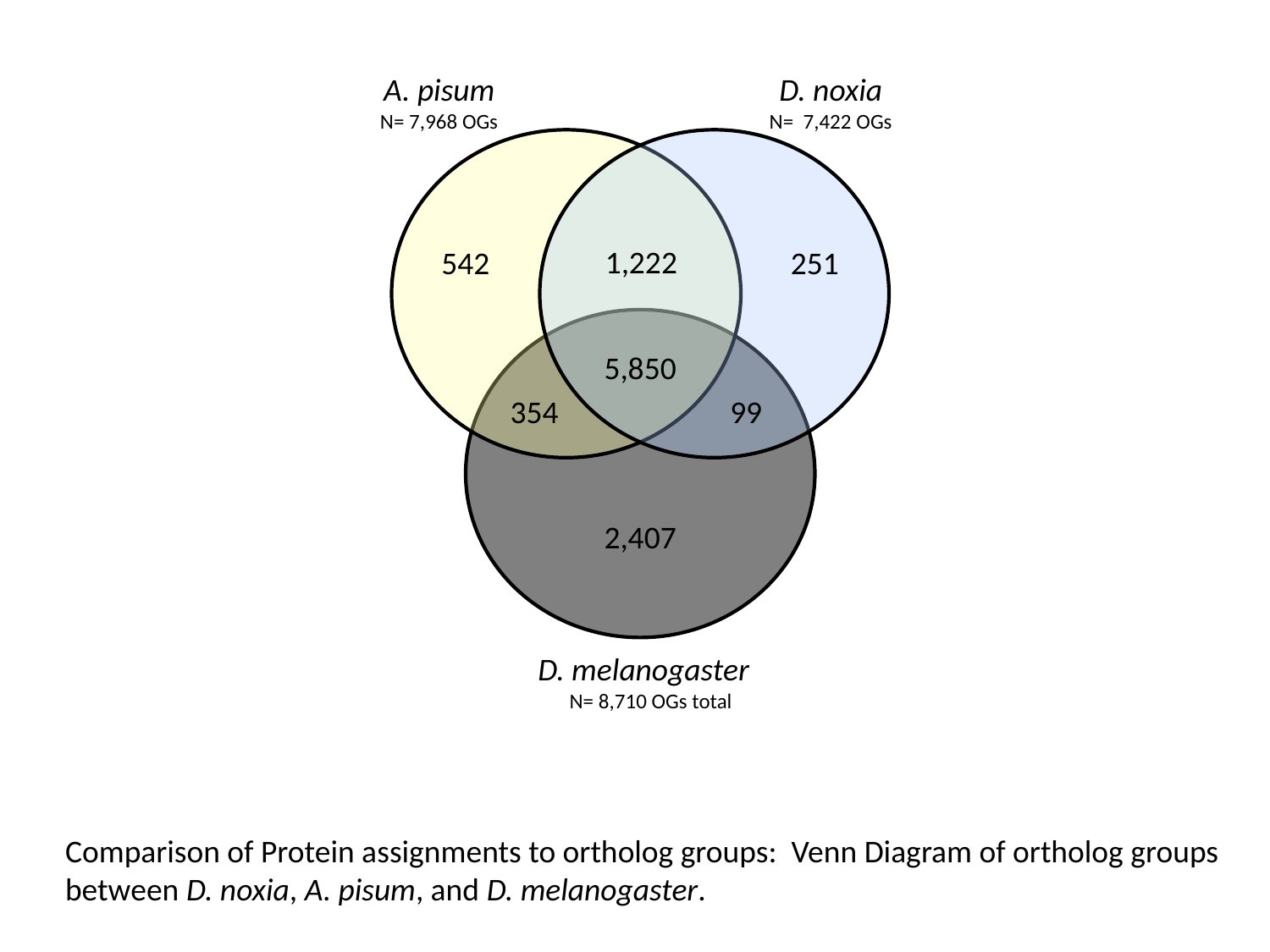

A. pisum
N= 7,968 OGs
D. noxia
N= 7,422 OGs
1,222
542
251
5,850
354
99
2,407
D. melanogaster
N= 8,710 OGs total
Comparison of Protein assignments to ortholog groups: Venn Diagram of ortholog groups between D. noxia, A. pisum, and D. melanogaster.

## Slide 6
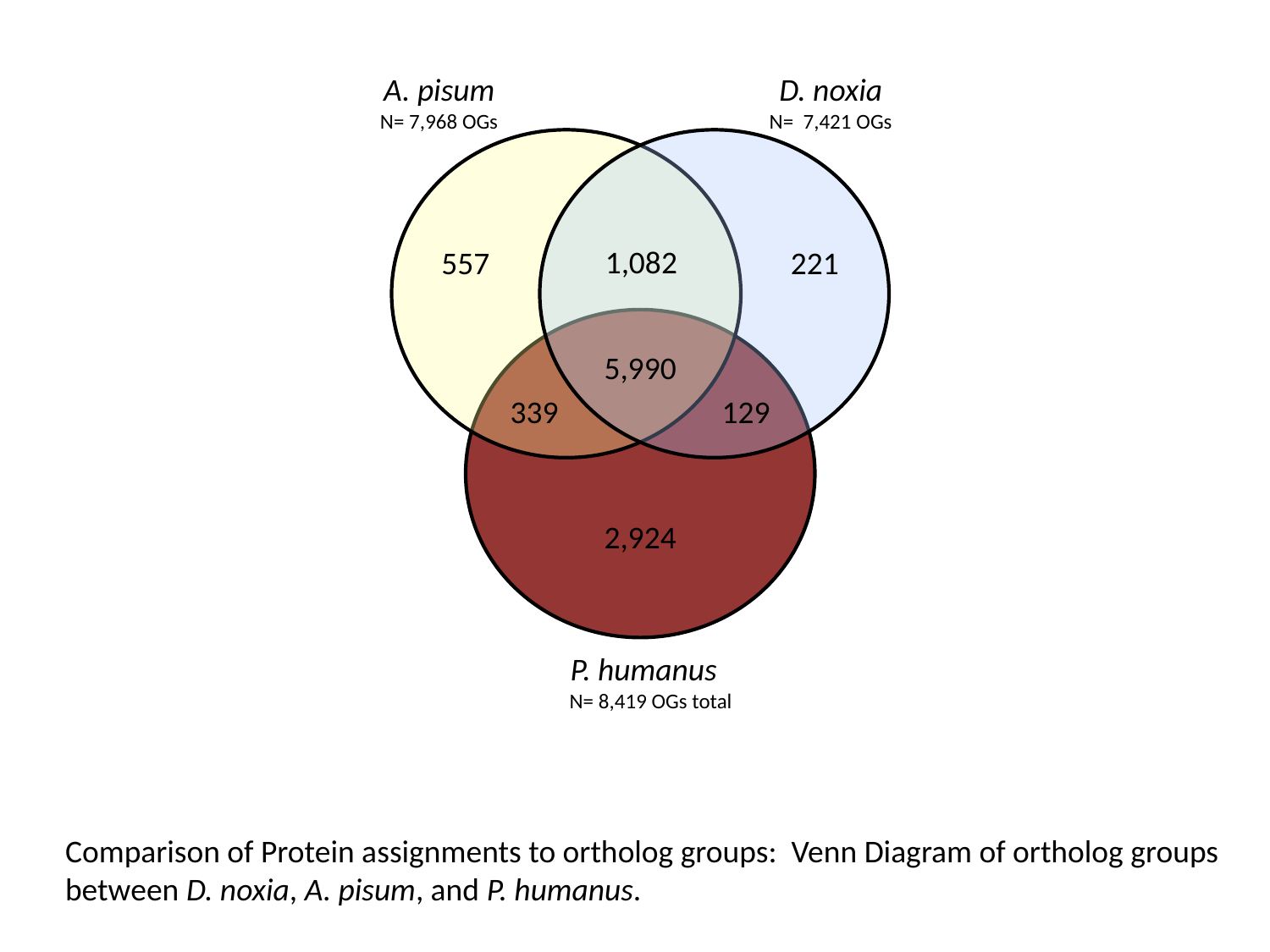

A. pisum
N= 7,968 OGs
D. noxia
N= 7,421 OGs
1,082
557
221
5,990
339
129
2,924
P. humanus
N= 8,419 OGs total
Comparison of Protein assignments to ortholog groups: Venn Diagram of ortholog groups between D. noxia, A. pisum, and P. humanus.

## Slide 7
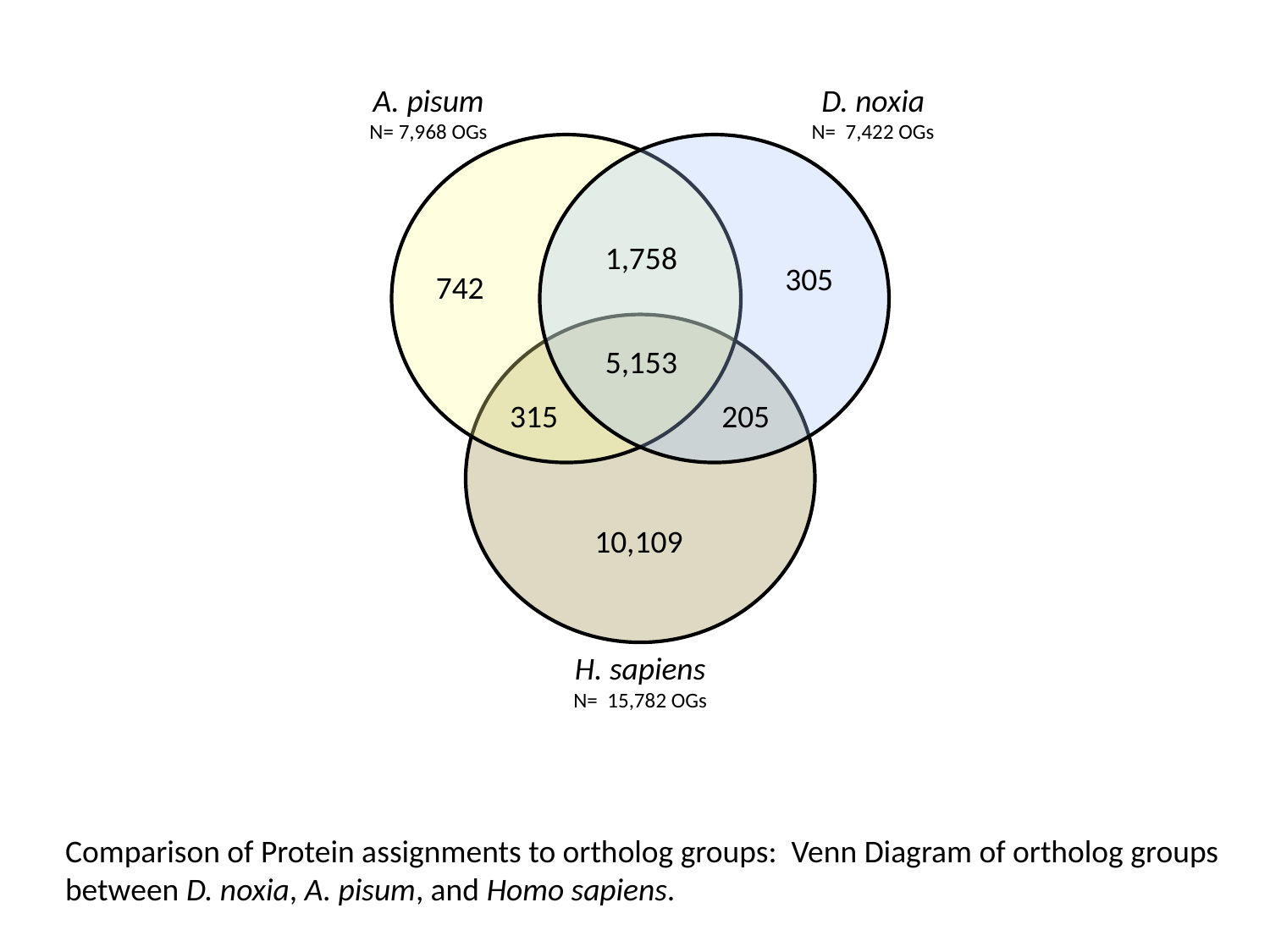

A. pisum
N= 7,968 OGs
D. noxia
N= 7,422 OGs
1,758
305
742
5,153
315
205
10,109
H. sapiens
N= 15,782 OGs
Comparison of Protein assignments to ortholog groups: Venn Diagram of ortholog groups between D. noxia, A. pisum, and Homo sapiens.

## Slide 8
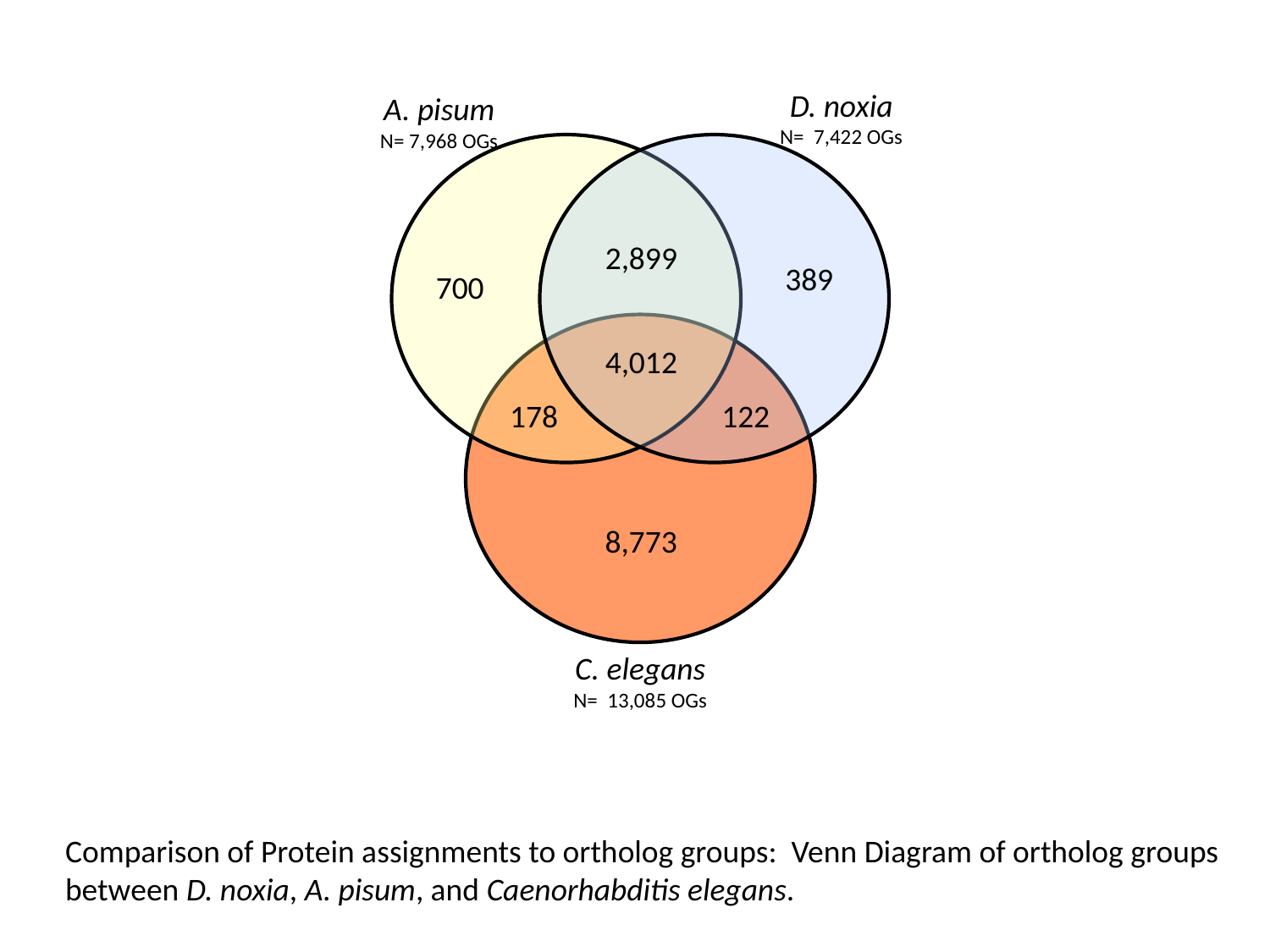

D. noxia
N= 7,422 OGs
A. pisum
N= 7,968 OGs
2,899
389
700
4,012
178
122
8,773
C. elegans
N= 13,085 OGs
Comparison of Protein assignments to ortholog groups: Venn Diagram of ortholog groups between D. noxia, A. pisum, and Caenorhabditis elegans.

## Slide 9
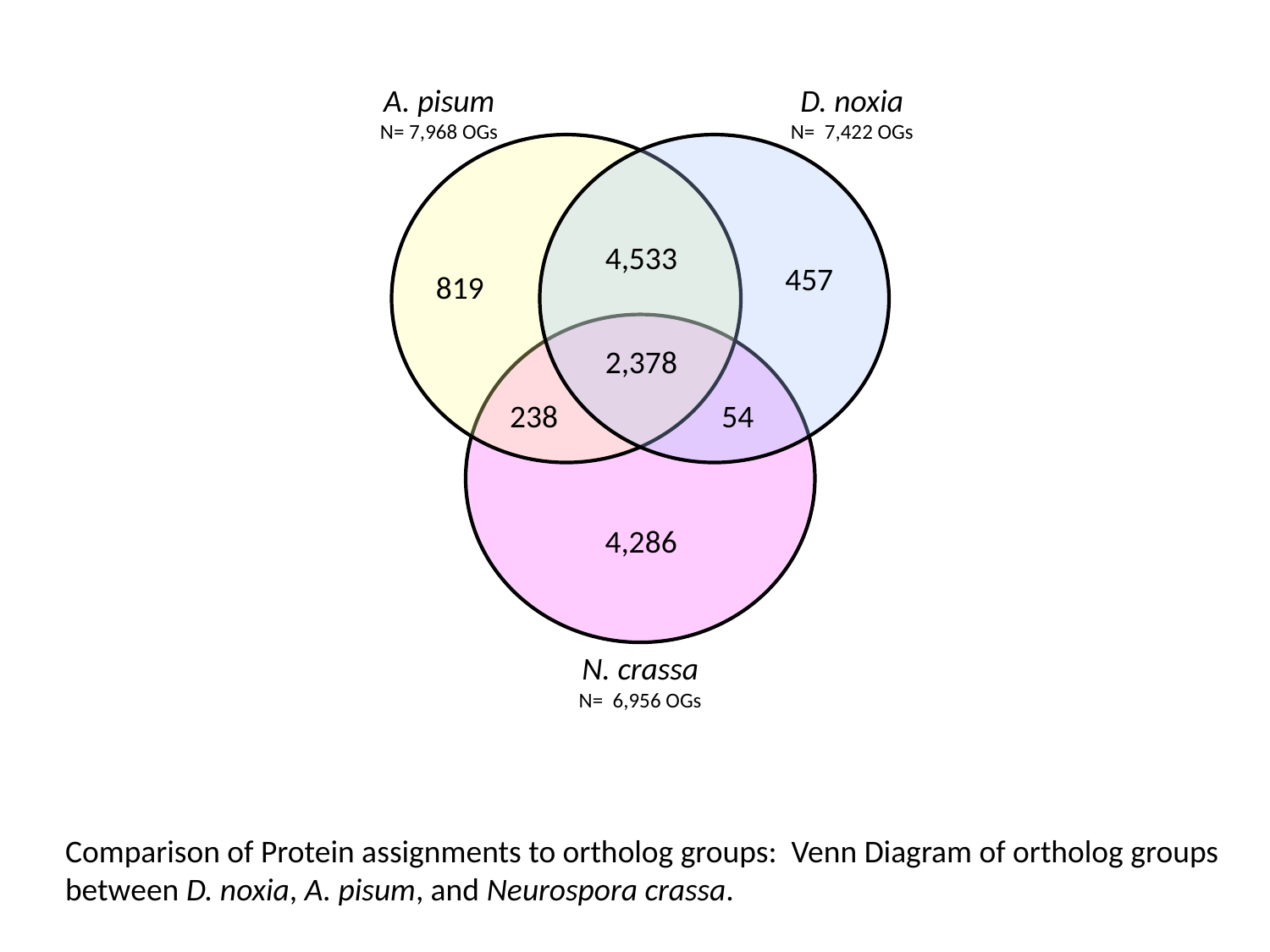

A. pisum
N= 7,968 OGs
D. noxia
N= 7,422 OGs
4,533
457
819
2,378
238
54
4,286
N. crassa
N= 6,956 OGs
Comparison of Protein assignments to ortholog groups: Venn Diagram of ortholog groups between D. noxia, A. pisum, and Neurospora crassa.

## Slide 10
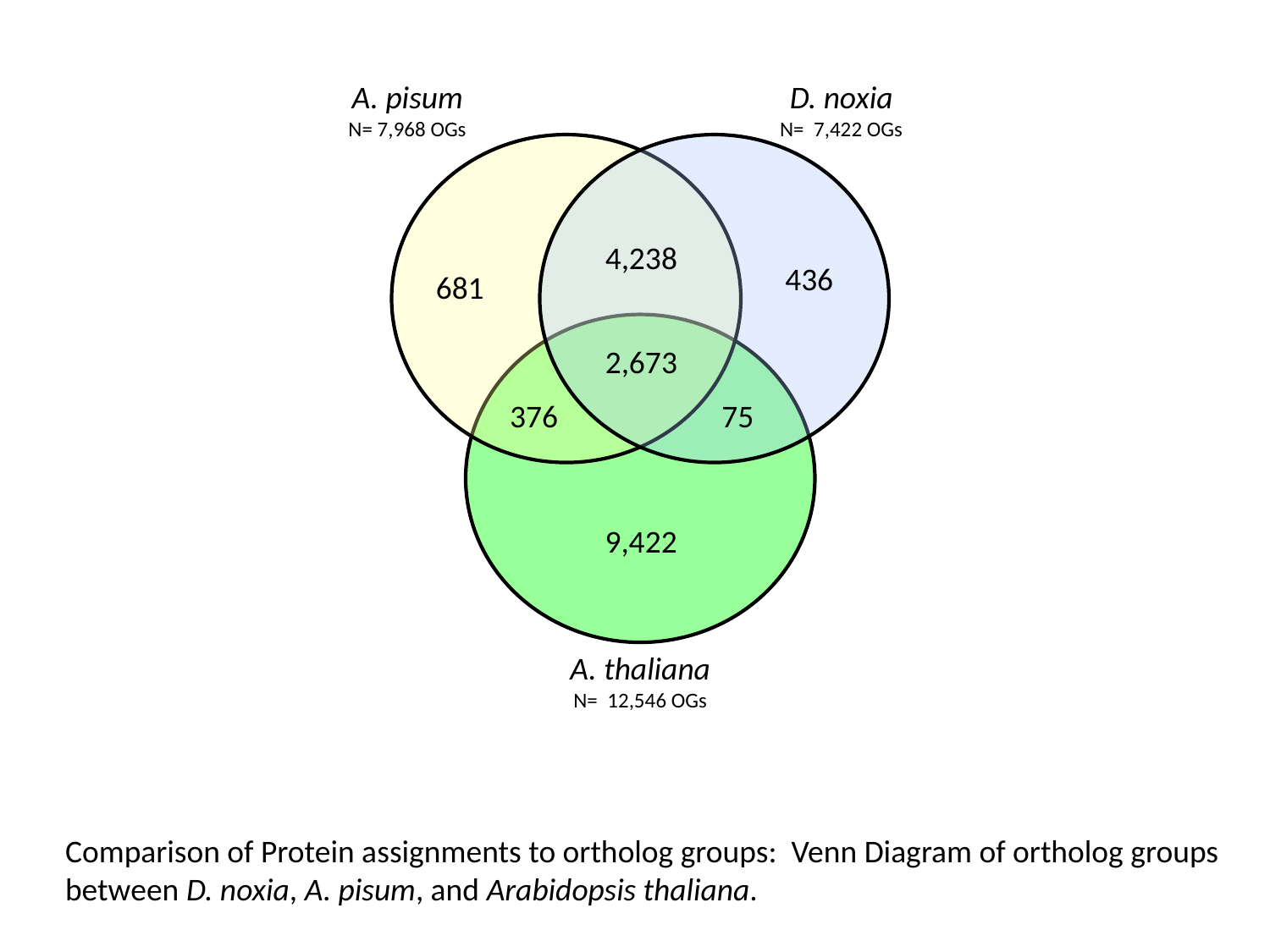

A. pisum
N= 7,968 OGs
D. noxia
N= 7,422 OGs
4,238
436
681
2,673
376
75
9,422
A. thaliana
N= 12,546 OGs
Comparison of Protein assignments to ortholog groups: Venn Diagram of ortholog groups between D. noxia, A. pisum, and Arabidopsis thaliana.
