# Supplementary material for: The genome of Diuraphis noxia, a global aphid pest of small grains
Source: BMC Genomics. 2015 Jun 5;16:429. doi: 10.1186/s12864-015-1525-1 (PMC4561433; doi:10.1186/s12864-015-1525-1)
Supplement: Additional file 17: Figure S4. — Phylogeny of apolipophorin among selected species. [file 12864_2015_1525_MOESM17_ESM.pptx]

## Slide 1
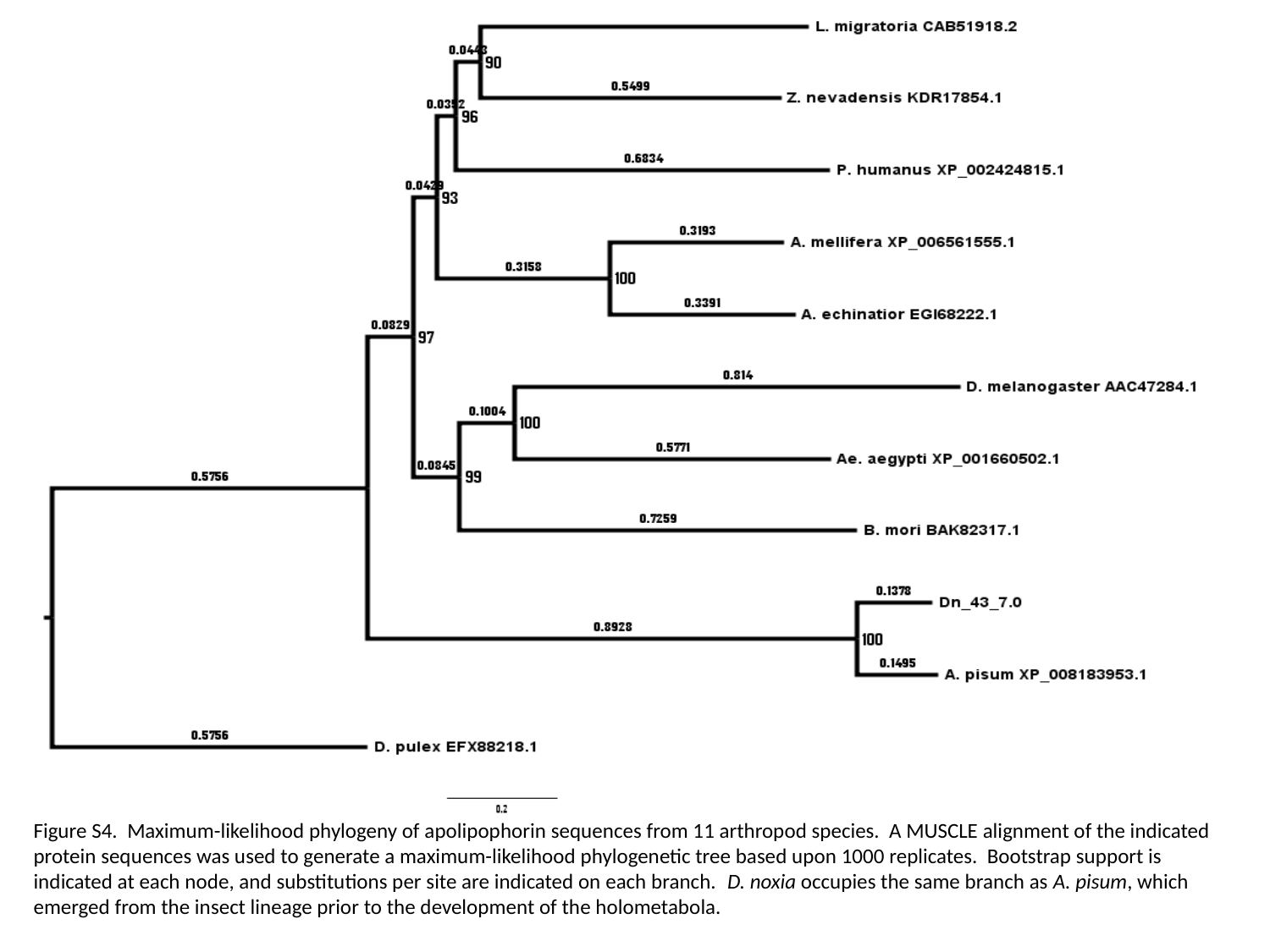

Figure S4. Maximum-likelihood phylogeny of apolipophorin sequences from 11 arthropod species. A MUSCLE alignment of the indicated protein sequences was used to generate a maximum-likelihood phylogenetic tree based upon 1000 replicates. Bootstrap support is indicated at each node, and substitutions per site are indicated on each branch. D. noxia occupies the same branch as A. pisum, which emerged from the insect lineage prior to the development of the holometabola.
